# Coordination Polymers from Biphenyl-Dicarboxylate Linkers: Synthesis, Structural Diversity, Interpenetration, and Catalytic Properties

**DOI:** 10.1021/acs.inorgchem.2c01488

**Published:** 2022-08-03

**Authors:** Xiaoyan Cheng, Lirong Guo, Hongyu Wang, Jinzhong Gu, Ying Yang, Marina V. Kirillova, Alexander M. Kirillov

**Affiliations:** † State Key Laboratory of Applied Organic Chemistry, Key Laboratory of Nonferrous Metal Chemistry and Resources Utilization of Gansu Province, College of Chemistry and Chemical Engineering, Lanzhou University, Lanzhou 730000, People’s Republic of China; ‡ Centro de Química Estrutural, Institute of Molecular Sciences, Departamento de Engenharia Química, Instituto Superior Técnico, Universidade de Lisboa, Av. Rovisco Pais, Lisbon 1049-001, Portugal

## Abstract

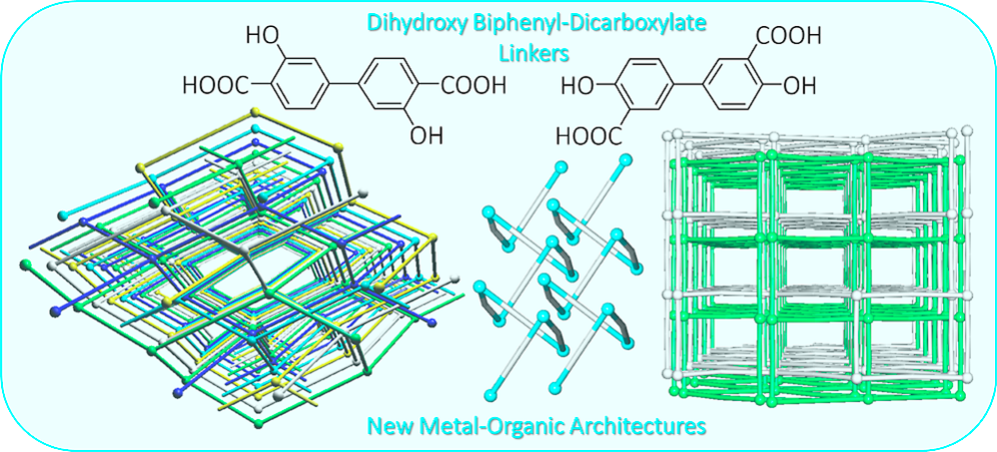

The present work explores two biphenyl-dicarboxylate linkers, 3,3′-dihydroxy-(1,1′-biphenyl)-4,4′-dicarboxylic (H_4_L_1_) and 4,4′-dihydroxy-(1,1′-biphenyl)-3,3′-dicarboxylic (H_4_L_2_) acids, in hydrothermal generation of nine new compounds formulated as [Co_2_(μ_2_-H_2_L_1_)_2_(phen)_2_(H_2_O)_4_] (**1**), [Mn_2_(μ_4_-H_2_L_1_)_2_(phen)_2_]_*n*_·4*n*H_2_O (**2**), [Zn(μ_2_-H_2_L_1_)(2,2′-bipy)(H_2_O)]_*n*_ (**3**), [Cd(μ_2_-H_2_L_1_) (2,2′-bipy)(H_2_O)]_*n*_ (**4**), [Mn_2_(μ_2_-H_2_L_1_)(μ_4_-H_2_L_1_)(μ_2_-4,4′-bipy)_2_]_*n*_·4*n*H_2_O (**5**), [Zn(μ_2_-H_2_L_1_)(μ_2_-4,4′-bipy)]_*n*_ (**6**), [Zn(μ_2_-H_2_L_2_)(phen)]_*n*_ (**7**), [Cd(μ_3_-H_2_L_2_)(phen)]_*n*_ (**8**), and [Cu(μ_2_-H_2_L_2_) (μ_2_-4,4′-bipy)(H_2_O)]_*n*_ (**9**). These coordination polymers (CPs) were generated by reacting a metal(II) chloride, a H_4_L_1_ or H_4_L_2_ linker, and a crystallization mediator such as 2,2′-bipy (2,2′-bipyridine), 4,4′-bipy (4,4′-bipyridine), or phen (1,10-phenanthroline). The structural types of **1**–**9** range from molecular dimers (**1**) to one-dimensional (**3**, **4**, **7**) and two-dimensional (**8**, **9**) CPs as well as three-dimensional metal–organic frameworks (**2**, **5**, **6**). Their structural, topological, and interpenetration features were underlined, including an identification of unique two- and fivefold 3D + 3D interpenetrated nets in **5** and **6**. Phase purity, thermal and luminescence behavior, as well as catalytic activity of the synthesized products were investigated. Particularly, a Zn(II)-based CP **3** acts as an effective and recyclable heterogeneous catalyst for Henry reaction between a model substrate (4-nitrobenzaldehyde) and nitroethane to give β-nitro alcohol products. For this reaction, various parameters were optimized, followed by the investigation of the substrate scope. By reporting nine new compounds and their structural traits and functional properties, the present work further outspreads a family of CPs constructed from the biphenyl-dicarboxylate H_4_L_1_ and H_4_L_2_ linkers.

## Abstract 2

Nine new metal−organic architectures were hydrothermally assembled from dihydroxy-functionalized biphenyl dicarboxylic acid linkers and were fully characterized. Their crystal structures, topology, and interpenetration features as well as catalytic behavior in the Henry reaction are highlighted.

## Introduction

Coordination polymers (CPs) and their porous subclass, well-known as MOFs (metal–organic frameworks), are currently of massive attention among researchers in areas of chemistry, physics, and material science.^1−6^ Such a tremendous recognition of these metal–organic architectures is largely governed by their infinite diversity of structural types and enthralling functional properties and applications in a multitude of research areas, including storage and separation of gases,^7−13^ sensing, luminescent and biomaterials,^14−19^ and catalysis,^20−26^ just to name a few.

Among a large variety of factors that may influence the assembly of CPs/MOFs,^27,28^ the selection of a principal building block acting as a linker and its intrinsic characteristics represent a central parameter.^29,30^ In addition, synthetic methods and reaction conditions can affect structural features and properties of the resulting compounds.^31−39^ In this regard, hydrothermal synthesis stands out as one of the most promising and useful methodologies, owing to a blend of intrinsic pressure and temperature parameters for crystallizing the products and using H_2_O as a green reaction medium.^40−43^

Multicarboxylic acids with aromatic cores possess great thermal stability and are the key building blocks for assembling CPs via hydrothermal synthesis.^29,44^ An attractive use of these types of organic linkers in the field of CPs/MOFs is attributed to their rich coordination chemistry, different p*K*_a_ values, aqueous solubility in the form of reactive salt derivatives, and attractive physicochemical characteristics.^20,21,31,33,34,39,45,46^

**Scheme 1:**
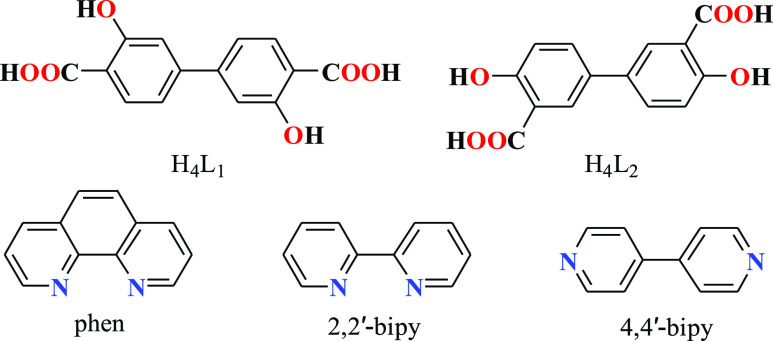
Formulae of H_4_L_1_ and H_4_L_2_ Linkers and *N*-Donor Crystallization Mediators

As an exploration of recent research of our groups in the field of hydrothermal preparation of novel metal–organic architectures from multicarboxylate linkers, we devoted our attention to hydroxy functionalized biphenyl-dicarboxylate building blocks, namely, 3,3′-dihydroxy-(1,1′-biphenyl)-4,4′-dicarboxylic (H_4_L_1_) and 4,4′-dihydroxy-(1,1′-biphenyl)-3,3′-dicarboxylic (H_4_L_2_) acids (Scheme 1).^47−52^ The selection of these carboxylic acids was governed by the following points: (i) both H_4_L_1_ and H_4_L_2_ are positional isomers with different locations of two COOH and two OH groups, (ii) the presence of biphenyl functionality permits some rotation along the carbon–carbon single bond, and (iii) both linkers are stable under hydrothermal conditions and can assume numerous modes of coordination. Given the extraordinary structural features and applications of some CPs/MOFs assembled from these types of ligands,^53−64^ the main aim of the current study consisted in further exploring H_4_L_1_ and H_4_L_2_ as promising dicarboxylate linkers for generating new types of CPs, followed by the investigation of their structural traits and some functional properties.

Thus, the present study describes the preparation procedures, structural characterization, topology and interpenetration features, luminescence properties, thermal stability, and catalytic behavior (Henry reaction) of new metal–organic architectures prepared from H_4_L_1_ or H_4_L_2_ and supporting ligands that mediate the crystallization (Scheme 1). The generated compounds have the following formulae: [Co_2_(μ_2_-H_2_L_1_)_2_(phen)_2_(H_2_O)_4_] (**1**), [Mn_2_(μ_4_-H_2_L_1_)_2_(phen)_2_]_*n*_·4*n*H_2_O (**2**), [Zn(μ_2_-H_2_L_1_)(2,2′-bipy)(H_2_O)]_*n*_ (**3**), [Cd(μ_2_-H_2_L_1_)(2,2′-bipy)(H_2_O)]_*n*_ (**4**), [Mn_2_(μ_2_-H_2_L_1_)(μ_4_-H_2_L_1_)(μ_2_-4,4′-bipy)_2_]_*n*_·4*n*H_2_O (**5**), [Zn(μ_2_-H_2_L_1_)(μ_2_-4,4′-bipy)]_*n*_ (**6**), [Zn(μ_2_-H_2_L_2_)(phen)]_*n*_ (**7**), [Cd(μ_3_-H_2_L_2_)(phen)]_*n*_ (**8**), and [Cu(μ_2_-H_2_L_2_)(μ_2_-4,4′-bipy)(H_2_O)]_*n*_ (**9**). These metal–organic architectures further broaden to new types of growing family of functional CPs generated from the biphenyl-dicarboxylate H_4_L_1_or H_4_L_2_ linkers.

## Experimental Section

### Brief Details on Synthesis of **1–9**

Commercially acquired reagents and solvents were used (AR grade). All compounds (**1**–**9**) were synthesized hydrothermally using different compositions of the reaction mixtures in water, which are summarized in Table 1. These mixtures were treated for 72 h at 160 °C, followed by steady cooling for product crystallization with a rate of 10 °C h^–1^. Detailed analytical data and synthesis procedures for each compound are given in the Supporting Information.

**Table 1:** Description of Hydrothermal Reaction Mixtures along with a Summary of Structural Features for Compounds **1**–**9**^a^

compound	metal chloride precursor	supporting ligand as a mediator of crystallization (MC)	dimensionality	topology
[Co_2_(μ_2_-H_2_L_1_)_2_(phen)_2_(H_2_O)_4_] (**1**)	CoCl_2_·6H_2_O	phen	0D	
[Mn_2_(μ_4_-H_2_L_1_)_2_(phen)_2_]_*n*_·4*n*H_2_O (**2**)	MnCl_2_·4H_2_O	phen	3D	**pts**
[Zn(μ_2_-H_2_L_1_)(2,2′-bipy)(H_2_O)]_*n*_ (**3**)	ZnCl_2_	2,2′-bipy	1D	2C1
[Cd(μ_2_-H_2_L_1_)(2,2′-bipy)(H_2_O)]_*n*_ (**4**)	CdCl_2_·H_2_O	2,2′-bipy	1D	2C1
[Mn_2_(μ_2_-H_2_L_1_)(μ_4_-H_2_L_1_)(μ_2_-4,4′-bipy)_2_]_*n*_·4*n*H_2_O (**5**)	MnCl_2_·4H_2_O	4,4′-bipy	3D + 3D^b^	**sqc65**
[Zn(μ_2_-H_2_L_1_)(μ_2_-4,4′-bipy)]_*n*_ (**6**)	ZnCl_2_	4,4′-bipy	3D + 3D^c^	**dia**
[Zn(μ_2_-H_2_L_2_)(phen)]_*n*_ (**7**)	ZnCl_2_	phen	1D	2C1
[Cd(μ_3_-H_2_L_2_)(phen)]_*n*_ (**8**)	CdCl_2_·H_2_O	phen	2D	**utp**
[Cu(μ_2_-H_2_L_2_)(μ_2_-4,4′-bipy)(H_2_O)]_*n*_ (**9**)	CuCl_2_·2H_2_O	4,4′-bipy	2D	**hcb**

a Reactions were carried out under hydrothermal settings: stainless steel autoclave (25 mL volume with teflon lining), H_2_O (10 mL), M^2+^/H_4_L/MC/NaOH molar ratio (1:1:1:2), 160 °C, 72 h.

b Twofold interpenetrated nets.

c Fivefold interpenetrated nets.

### X-ray Diffraction

For single crystals of **1**–**9**, the X-ray data were obtained on a Bruker Smart CCD or an Agilent SuperNova diffractometer (graphite-monochromated Mo K_α_ radiation, λ = 0.71073 Å). Semiempirical absorption correction was performed with SADABS, whereas SHELXS-97/SHELXL-97^65,66^ was applied for solving (direct methods) and refining (full-matrix least-squares on *F*^2^) the structures. The non-hydrogen atoms were refined anisotropically (full-matrix least-squares on *F*^2^), while the carbon-bound hydrogens were added to calculated positions with fixed isotropic thermal parameters. In COOH/H_2_O moieties, hydrogen atoms were placed using difference maps and constrained to the respective parent oxygen atoms. In **2** and **5**, some very disordered solvent molecules were eliminated by applying SQUEEZE in PLATON.^67^ The amount of crystallization molecules of solvent was calculated based on C/H/N and TGA analyses. In **4**, the OH group of H_2_L_1_^2–^ was split over two sites and refined with 0.60 and 0.40 occupancies. In **5**, the OH moiety of H_2_L_1_^2–^ was also split over two sites and refined with occupancies of 0.384 and 0.616. In **6**, the disordered aromatic ring of H_2_L_1_^2–^ was refined with equal occupancies. A summary of crystal data for all the structures is provided in Table 2. Representative bonding parameters (Table S1) and hydrogen bond data (Table S2) are given in the Supporting Information.

**Table 2:** Crystallographic Data for Compounds **1**–**9**

compound	1	2	3	4	5
chemical formula	C_26_H_20_CoN_2_O_8_	C_52_H_40_Mn_2_N_4_O_16_	C_24_H_18_ZnN_2_O_7_	C_24_H_18_CdN_2_O_7_	C_24_H_19_MnN_2_O_8_
formula weight	547.37	1086.69	511.77	558.80	518.32
crystal system	Triclinic	triclinic	monoclinic	orthorhombic	monoclinic
space group	*P*1̅	*P*1̅	*I*2/*a*	*Pcab*	*P*1_2_/*c*1
*a*/Å	7.51048(13)	14.04807(16)	18.4131(2)	6.93160(10)	14.4043(4)
*b*/Å	10.16251(14)	14.08253(15)	7.82310(10)	23.5275(3)	11.6030(3)
*c*/Å	15.9746(2)	14.09923(17)	30.0763(4)	28.1740(3)	17.3938(5)
α/°	101.6072(12)	61.7498(12)	90	90	90
β/°	101.8231(13)	79.7349(11)	96.0530(10)	90	107.851(3)
γ/°	99.9253(13)	73.1773(10)	90	90	90
*V*/Å^3^	1139.62(3)	2349.09(5)	4308.26(9)	4594.71(10)	2767.12(14)
*T*/K	293(2)	293(2)	293(2)	293(2)	293(2)
*Z*	2	2	8	8	4
*D*_c_/g cm^–3^	1.595	1.435	1.578	1.616	1.158
μ/mm^–1^	6.411	4.958	2.030	8.034	4.183
*F*(000)	562	1036	2096	2240	984
refl. measured	14270	35015	14701	15495	16555
unique refl. (*R*_int_)	4211 (0.0338)	8689 (0.0486)	3993 (0.0251)	4160 (0.0294)	5345 (0.0920)
GOF on *F*^2^	1.044	1.008	1.087	1.042	1.107
*R*_1_ [*I* > 2σ(*I*)]	0.0315	0.0403	0.0371	0.0288	0.0844
*wR*_2_ [*I* > 2σ(*I*)]	0.0804	0.1021	0.1090	0.0807	0.1039

compound	**6**	**7**	**8**	**9**
chemical formula	C_24_H_16_ZnN_2_O_6_	C_26_H_16_ZnN_2_O_6_	C_26_H_16_CdN_2_O_6_	C_24_H_17_CuN_2_O_7_
formula weight	493.76	517.78	564.81	508.93
crystal system	triclinic	orthorhombic	orthorhombic	triclinic
space group	*P*1̅	*Pna*2_1_	*Pna*2_1_	*P*1̅
*a*/Å	8.86840(10)	13.0069(4)	10.1025(4)	8.6127(6)
*b*/Å	11.0591(2)	15.7790(5)	10.2188(4)	9.3670(6)
*c*/Å	11.96730(10)	10.7000(4)	20.7971(8)	13.4134(6)
α/°	85.4940(10)	90	90	99.604(5)
β/°	76.8250(10)	90	90	94.884(5)
γ/°	71.8150(10)	90	90	99.304(6)
*V*/Å^3^	1085.70(3)	2196.01(13)	2147.00(15)	1045.91(11)
*T*/K	293(2)	293(2)	293(2)	293(2)
*Z*	2	4	4	2
*D*_c_/g cm^–3^	1.510	1.566	1.747	1.616
μ/mm^–1^	1.958	1.969	8.573	1.934
*F*(000)	504	1056	1128	520
refl. measured	10798	8426	2755	6468
unique refl. (*R*_int_)	4249 (0.0376)	3044 (0.0417)	2328 (0.0398)	3324 (0.0431)
GOF on *F*^2^	1.172	1.079	1.045	1.053
*R*_1_ [*I* > 2σ(*I*)]	0.0686	0.0350	0.0351	0.0716
*wR*_2_ [*I* > 2σ(*I*)]	0.0709	0.0411	0.0477	0.1095

In the obtained crystal structures, metal–organic (**2**–**9**) or H-bonded (**1**) networks were analyzed topologically by following a concept of underlying (simplified) network.^68,69^ To obtain simplified metal–organic or H-bonded networks, the bridging ligands or molecular units were contracted, respectively, to the centroids while maintaining their connectivities.^55,56^ CCDC2096490–2096498 enclose the crystallographic parameters of **1**–**9**.

### Henry Reaction

Under typical conditions, a reaction mixture contained in a capped glass vessel and composed of 4-nitrobenzaldehyde (0.50 mmol; model substrate), nitroethane (2.0 mmol), and catalyst (4 mol %) in methanol (1.0 mL) was stirred for 12 h at 70 °C. The catalyst was then isolated through centrifuging the reaction mixture. The obtained solution was subjected to evaporation in vacuo to form a crude product. Its part was dissolved in deuterated chloroform for subsequent analysis by ^1^H NMR spectroscopy using a JNM ECS 400 M spectrometer (for details, see Figure S4, Supporting Information). To carry out the catalyst recycling tests, after each reaction step, the catalyst was centrifuged, washed by methanol, desiccated, and used in the next cycle. Successive reaction cycles were accomplished as mentioned above. Various blank tests were run to confirm the importance of coordination compounds as catalysts. Effects of various reaction conditions such as temperature, time, solvent, and substrate scope were also investigated.

## Results and Discussion

### Hydrothermal Synthesis of **1–9**

Both carboxylic acid building blocks, 3,3′-dihydroxy-(1,1′-biphenyl)-4,4′-dicarboxylic acid (H_4_L_1_) and 4,4′-dihydroxy-(1,1′-biphenyl)-3,3′-dicarboxylic acid (H_4_L_2_), represent up to six potential O-sites for coordination. To further explore their application toward the design of novel CPs/MOFs, several hydrothermal reactions were attempted using aqueous mixtures composed of M(II) chlorides [M(II) = Co, Mn, Zn, Cd, Cu], H_4_L_1_ or H_4_L_2_ as a linker, NaOH as a base, and a series of crystallization mediators (Scheme 1). These auxiliary ligands were 2,2′-bipy(2,2′-bipyridine), 4,4′-bipy(4,4′-bipyridine), or phen(1,10-phenanthroline), Among various reactions attempted, nine hydrothermal syntheses were well reproducible and permitted an isolation of pure crystalline product fractions (Table 1) that also contained monocrystals appropriate for X-ray diffraction study. In contrast to H_4_L_1_, the products derived from H_4_L_2_ turned to be more difficult to crystallize, and we were able to isolate only three compounds (**7**–**9**). The manganese(II) CP **2** and MOF **5** reveal different structural types owing to different crystallization mediators (phen for **2** or 4,4′-bipy for **5**); in the latter case, the μ_2_-4,4′-bipy acts as an additional linker that is responsible for increasing the dimensionality to a 3D net. Similarly, the structures of Zn(II) CPs **3** and **6** are also affected by the type of crystallization mediator, revealing 1D chains or 3D frameworks, respectively. CPs **5** and **6** were synthesized using the same procedure but altering the metal(II) precursor (MnCl_2_ for **5** and ZnCl_2_ for **6**), resulting in 3D interpenetrated nets in both cases but with different topologies and degrees of interpenetration. The structures of **7** and **8** are also affected by the type of metal precursor used, despite the similarity of other reaction conditions. With regard to the type of biphenyl-dicarboxylate linker, the direct comparison of the isolated products is difficult as not all synthetic attempts resulted in the isolation of pure crystalline products. For example, when using the same crystallization mediator (phen), the products derived from H_4_L_1_ were isolated in the case of Co(II) and Mn(II), while similar reactions with H_4_L_2_ permitted to crystallize only zinc(II) and cadmium(II) derivatives (Table 1). Nevertheless, zinc(II) derivatives **3** and **7** with closely related supporting ligands (2,2′-bipy and phen) feature similar types of 1D CP structures. In contrast, the Cd(II) products **4** (1D CP) and **8** (2D CP) show a difference in their dimensionality and topology that is likely influenced by the type of biphenyl–dicarboxylate linker.

**Scheme 2:**
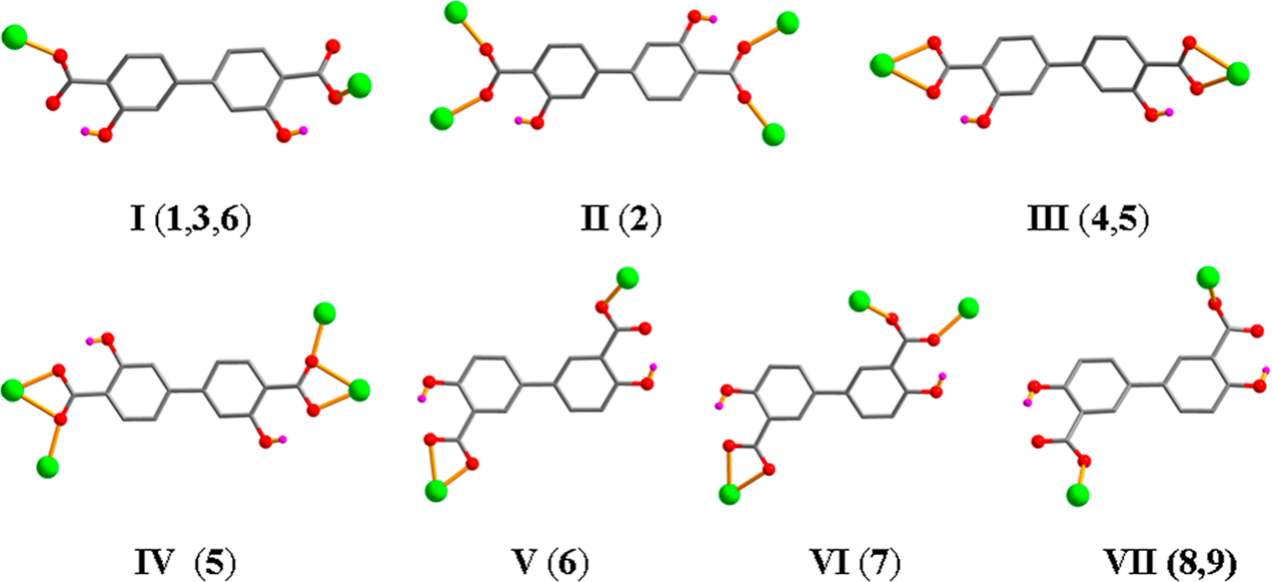
Coordination Modes for H_2_L_1_^2–^ (Modes I–IV) and H_2_L_2_^2–^ (Modes V–VII) in Structures of **1**–**9**

In general, structural differences in **1**–**9** indicate that their metal–organic architectures depend on crystallization mediator, metal precursor, and main dicarboxylic acid linker (with COOH and OH groups in different positions of the biphenyl core). The biphenyl–dicarboxylate linkers exhibit up to seven distinct coordination modes (Scheme 2) with COO^–^ groups of different denticities. Also, the H_2_L_1_^2–^/H_2_L_2_^2–^ linkers in **1**–**9** feature a rotation of the rings along the C–C bond with the corresponding angles (dihedral) ranging from 0.0 to 46.21°, thus enabling an adjustment of ligands to coordination preferences of metal ions. All new compounds were fully characterized in the solid state, including the determination of crystal structures by X-ray diffraction (Table 2). The latter disclose molecular dimers (**1**), one-dimensional (**3**, **4**, **7**) and two-dimensional (**8**, **9**) CPs as well as three-dimensional (**2**, **5**, **6**) metal–organic networks.

### Structural Description

#### [Co_2_(μ_2_-H_2_L_1_)_2_(phen)_2_(H_2_O)_4_] (**1**)

**Figure 1:**
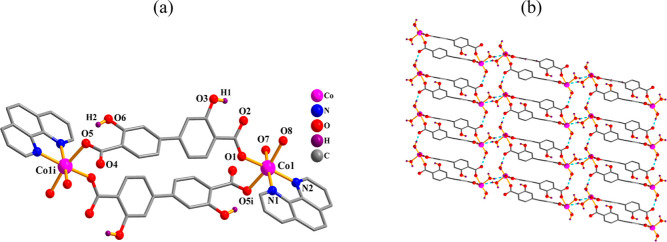
Crystal structure of compound **1**. (a) Co_2_ dimer; only OH hydrogen atoms are shown; (b) 2D H-bonded layer; phen ligands are not shown; representation along the *b*-axis.

The structure of **1** reveals a dimeric complex (Figure 1) that contains one Co(II) atom, one μ_2_-H_2_L_1_^2–^ linker, one phen ligand, and two terminal water ligands in the asymmetric entity. Both six-coordinate Co1 centers display a distorted {CoN_2_O_4_} octahedral environment that is constructed from two carboxylate oxygen atoms from two μ_2_-H_2_L_1_^2–^ blocks, a pair of N_phen_ atoms, and two water ligands. The distances of Co–N [2.123(2)–2.132(2) Å] and Co–O [2.042(2)–2.184(2) Å] bonds agree with typical literature data.^21,31,70^ Two H_2_L_1_^2–^ ligands act as μ_2_-linkers via monodentate carboxylate groups (Scheme 2, mode I), thus assembling two Co1 centers to give a cyclic Co_2_ complex with a Co···Co separation of 14.056(2) Å (Figure 1a). Such Co_2_ molecular units are involved in an intermolecular H-bonding forming a 2D network (Figure 1b) with an **sql** topology.^71,72^

#### [Mn_2_(μ_4_-H_2_L_1_)_2_(phen)_2_]_*n*_·4*n*H_2_O (**2**)

**Figure 2:**
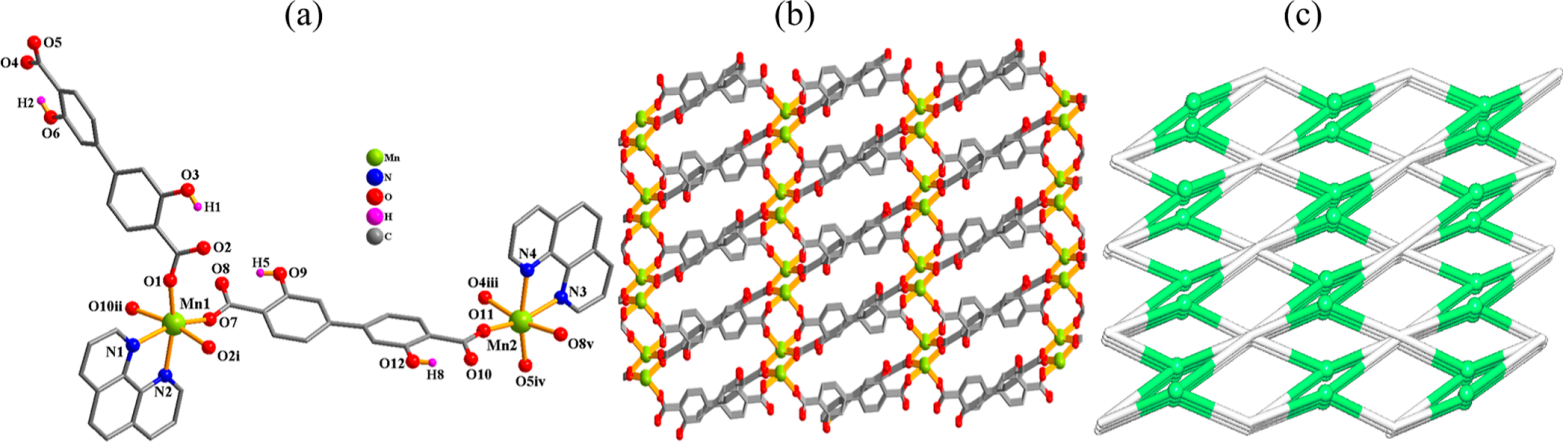
Crystal structure of **2**. (a) Connectivity and coordination environments of metal atoms; CH atoms are not shown. (b) 3D MOF; phen ligands are omitted; view along the *a*-axis. (c) Topological view of a dinodal 4,4-connected **pts** network; representation along the *a*-axis; centroids of 4-linked μ_4_-H_2_L_1_^2–^ nodes (gray); 4-linked Mn nodes (green balls).

An asymmetric unit of this 3D MOF contains two manganese(II) atoms, two μ_4_-H_2_L_1_^2–^ ligands, and two terminal phen moieties (Figure 2). The Mn1/Mn2 centers are six-coordinate and assume the distorted octahedral {MnN_2_O_4_} environments (Figure 2a). These are filled by four oxygen atoms from four μ_4_-H_2_L_1_^2–^ linkers and two N_phen_ donors. The distances of the Mn–N [2.301(2)–2.308(2) Å] and Mn–O [2.087(2)–2.209(2) Å] bonds are within typical values.^31,39,46^ The H_2_L_1_^2–^ ligands function as μ_4_-linkers with carboxylate functionalities adopting μ_2_-bridging bidentate modes (Scheme 2, mode II). These dicarboxylate linkers multiply sew the Mn(II) centers into a 3D MOF (Figure 2b). Topologically, MOF **2** is constructed from the 4-linked Mn(II) and μ_4_-H_2_L_1_ nodes, forming a dinodal 4,4-linked framework of a **pts** [PtS, Cooperite] topological type with a (4^2^.8^4^) point symbol (Figure 2c).^73^

#### [Zn(μ_2_-H_2_L_1_)(2,2′-bipy)(H_2_O)]_*n*_ (**3**)

**Figure 3:**
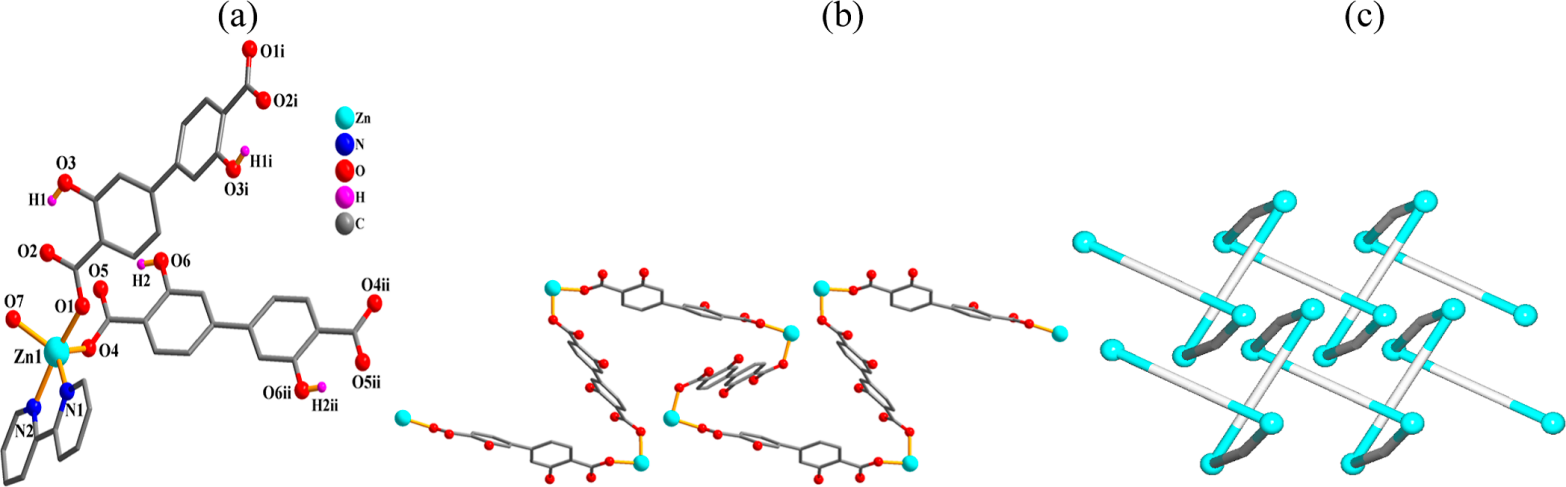
Crystal structure of **3**. (a) Connectivity and coordination environment of metal center; CH atoms are not shown. (b) Helical metal–organic chain; 2,2′-bipy ligands are omitted; representation along the *c*-axis. (c) Topological view of two helical 2C1 chains; centroids of μ_2_-H_2_L_1_^2–^ linkers (gray); Zn atoms (turquoise balls).

This CP reveals a 1D helical chain structure (Figure 3) composed of a zinc(II) center, two-halves of μ_2_-H_2_L_1_^2–^ linker, a 2,2′-bipy ligand, and a terminal water ligand per asymmetric unit. The Zn1 atom is five-coordinate and shows a distorted trigonal bipyramidal {ZnN_2_O_3_} environment (Figure 3a), which is taken by a pair of O donors from two μ_2_-H_2_L_1_^2–^ blocks, one water ligand, and a pair of N_2,2′-bipy_ atoms. The lengths of Zn–N [2.095(2)–2.113(2) Å] and Zn–O [1.990(2)–2.041(2) Å] bonds are standard for such type of compounds.^31,74,75^ The H_2_L_1_^2–^ block behaves as a μ-linker (Scheme 2, mode I), bridging the Zn1 centers into one-dimensional helical chains (Figure 3b) with a Zn1···Zn1 separation of 15.495(3) Å. These chains feature a 2C1 topology (Figure 3c).

#### [Cd(μ_2_-H_2_L_1_)(2,2′-bipy)(H_2_O)]_*n*_ (**4**)

**Figure 4:**
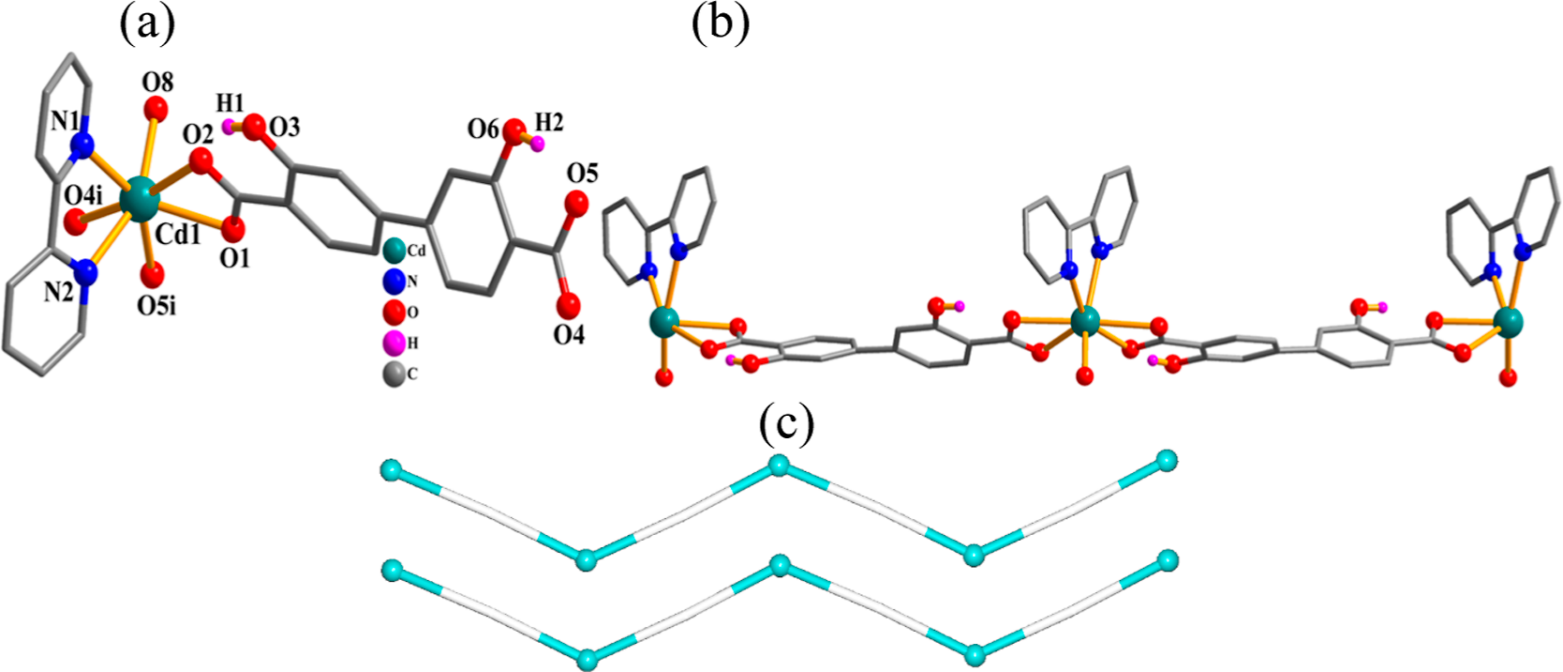
Crystal structure of **4**. (a) Connectivity and coordination environment of metal center; CH atoms are not shown. (b) 1D CP chain; representation along the *a*-axis. (c) Topological view of two 2C1 chains; centroids of μ_2_-H_2_L_1_^2–^ linkers (gray), Cd atoms (turquoise balls).

The one-dimensional CP **4** comprises a Cd1 atom, a μ_2_-H_2_L_1_^2–^ linker, a 2,2′-bipy moiety, and a water ligand per asymmetric unit (Figure 4). The Cd1 atom is seven-coordinate and possesses a distorted {CdN_2_O_5_} pentagonal bipyramidal environment. It is constructed from four carboxylate oxygen donors from a pair of μ_2_-H_2_L_1_^2–^ linkers, two N_2,2′-bipy_ atoms, and an H_2_O ligand (Figure 4a). The H_2_L_1_^2–^ ligand assumes a μ_2_-coordination mode with bidentate carboxylate functionalities (Scheme 2, mode III). The H_2_L_1_^2–^ linkers connect the Cd(II) atoms into 1D chains of a 2C1 topological type (Figure 4b,c).

#### [Mn_2_(μ_2_-H_2_L_1_)(μ_4_-H_2_L_1_)(μ_2_-4,4′-bipy)_2_]_*n*_·4*n*H_2_O (**5**)

**Figure 5:**
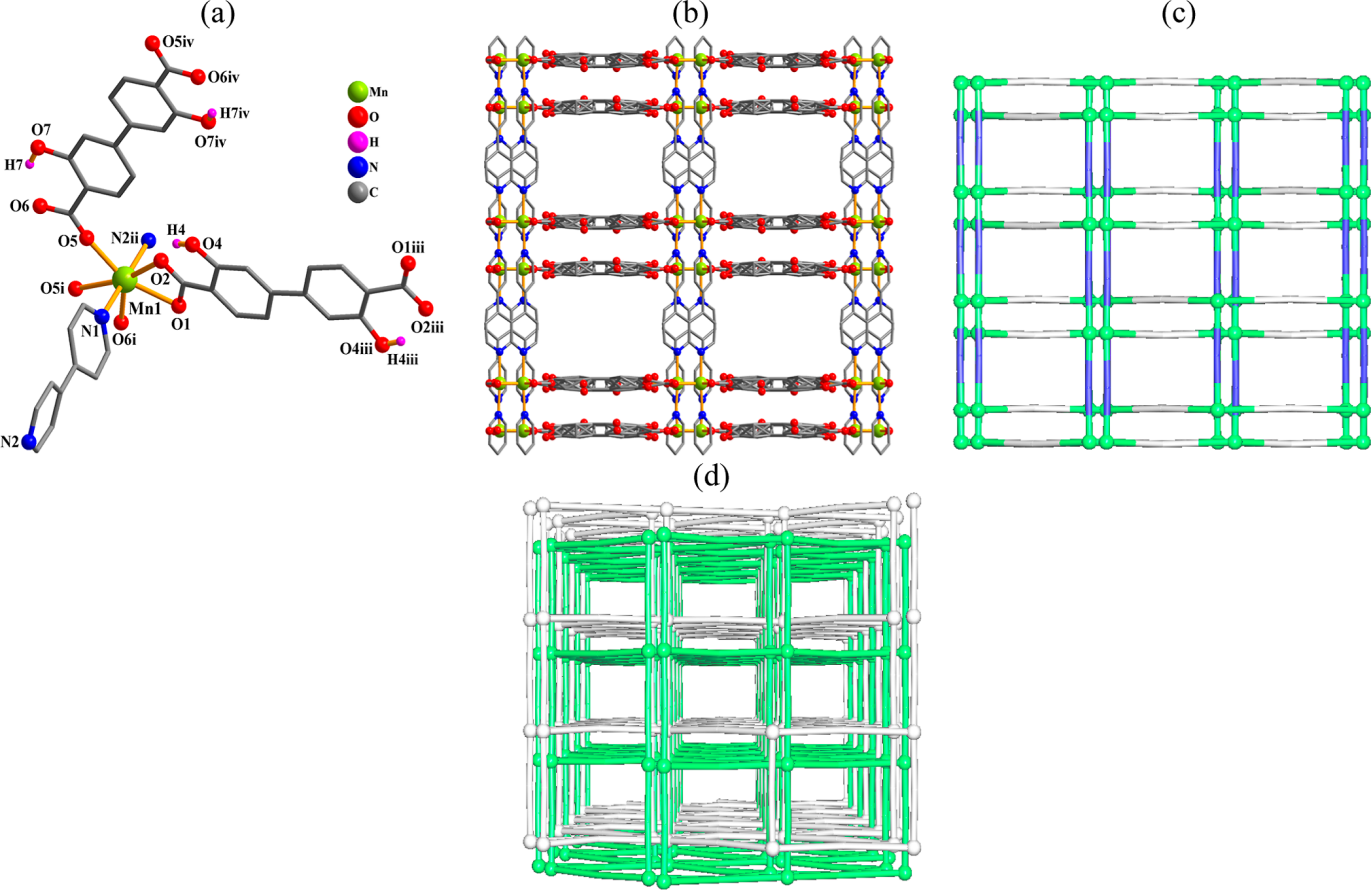
Crystal structure of **5**. (a) Connectivity and coordination environment metal atom; CH atoms are not shown. (b) Three-dimensional MOF; view along the *c*-axis. (c) Topological view of a dinodal 4,5-linked network with an **sqc65** topology; view along the *c*-axis; centroids of 2- and 4-linked μ_2_- and μ_4_-H_2_L_1_^2–^ nodes (gray), centroids of 2-linked μ_2_-4,4′-bipy moieties (blue), and 5-linked Mn nodes (green balls). (d) Perspective view of two interpenetrated frameworks represented by green and gray colors.

This compound possesses a 3D MOF structure (Figure 5). It comprises a Mn1 atom, a half of μ_2_-H_2_L_1_^2–^ and a half of μ_4_-H_2_L_1_^2–^ linkers, a μ_2_-4,4′-bipy moiety, and two crystallization water molecules in the asymmetric unit. The seven-coordinate Mn1 center reveals a distorted {MnN_2_O_5_} pentagonal bipyramidal fashion. The coordination sphere contains five oxygen atoms from three H_2_L_1_^2–^ linkers and two nitrogen atoms from two different 4,4′-bipy ligands (Figure 5a). The Mn–N [2.256(4)–2.259(4) Å] and Mn–O [2.212(3)–2.504(3) Å] distances well compare with typical values.^31,39,63^ The H_2_L_1_^2–^ blocks exhibit μ_2_- or μ_4_-coordination fashions with carboxylate moieties being bridging tridentate or bidentate (Scheme 2, modes IV and III). These dicarboxylate linkers, along with additional μ_2_-4,4′-bipy pillars, connect the Mn1 atoms into a three-dimensional MOF (Figure 5c). Topologically, the structure is composed of the 5-connected Mn nodes, 2- and 4-linked μ_2_- and μ_4_-H_2_L_1_^2–^ blocks, and 2-linked μ_2_-4,4′-bipy moieties. A binodal 4,5-connected network is generated (Figure 5c) and can be classified within an **sqc65** (epinet) type. It has a (4^2^.6^4^) (4^3^.6^7^)_2_ point symbol with the (4^2^.6^4^) and (4^3^.6^7^) notations referring to the μ_4_-H_2_L_1_^2–^ and Mn nodes, respectively. A remarkable peculiarity of **5** also consists in two interpenetrated networks (Figure 5d) having the parameters as follows: class IIa; total degree of interpenetration, *Z* = 2; interpenetration primitive cell, PIC: [1,0,0][0,0,1][0,1,0].

#### [Zn(μ_2_-H_2_L_1_)(μ_2_-4,4′-bipy)]_*n*_ (**6**)

**Figure 6:**
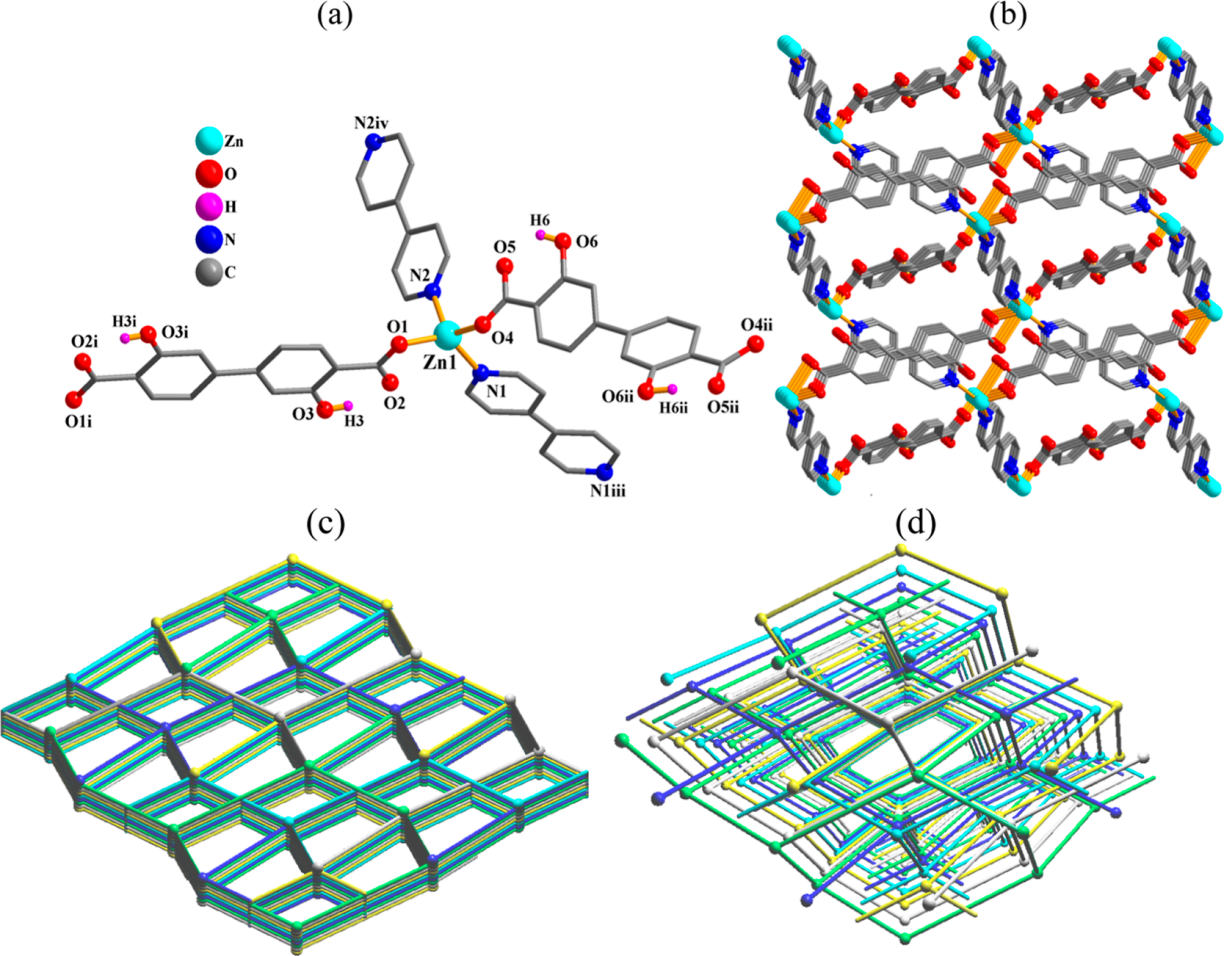
Crystal structure of **6**. (a) Connectivity and coordination environment of metal atom; CH atoms are not shown. (b) 3D MOF; view along the *a*-axis. (c) Topological view of a mononodal 4-linked **dia** network; representation along the *a*-axis; centroids of 2-linked μ_2_-H_2_L_1_^2–^ and μ_2_-4,4′-bipy moieties (sticks), 4-linked Zn nodes (balls). (d) Perspective view of five interpenetrated frameworks represented in green, blue, cyan, yellow, and gray colors.

This compound also shows an interpenetrated 3D MOF structure (Figure 6). Its asymmetric unit holds a Zn1 center, two halves of μ_2_-H_2_L_1_^2–^ ligands, and two halves of μ_2_-4,4′-bipy ligands. The four-coordinate Zn1 center unveils a trigonal pyramidal {ZnN_2_O_2_} coordination fashion with two carboxylate oxygen donors from a pair of μ_2_-H_2_L_1_^2–^ ligands and two nitrogen donors from two μ_2_-4,4′-bipy linkers (Figure 6a). The Zn–N [2.055(4)–2.059(5) Å] and Zn–O [1.922(4)–1.964(4) Å] bonds well agree with typical values for this type of compounds.^31,56,74^ The H_2_L_1_^2–^ ligands act as μ_2_-linkers (Scheme 2, mode I) with monodentate carboxylate functionalities. The μ_2_-H_2_L_1_^2–^ and μ_2_-4,4′-bipy ligands connect the Zn1 centers to produce a 3D MOF structure (Figure 6b). Topologically, the structure comprises the 4-linked Zn nodes and the 2-linked μ_2_-H_2_L_1_^2–^ and μ_2_-4,4′-bipy moieties (Figure 6c), generating a uninodal 4-connected net of the **dia** topological type with a point symbol of (6^6^). As in the case of **5**, compound **6** also features interpenetration, but remarkably, there are five 3D + 3D interpenetrated nets (Figure 6d) having the parameters as follows: class Ia; total degree of interpenetration, *Z* = 5; interpenetration primitive cell, PIC: [5,0,0][0,1,1][2,0,1].

#### [Zn(μ_2_-H_2_L_2_)(phen)]_*n*_ (**7**)

**Figure 7:**
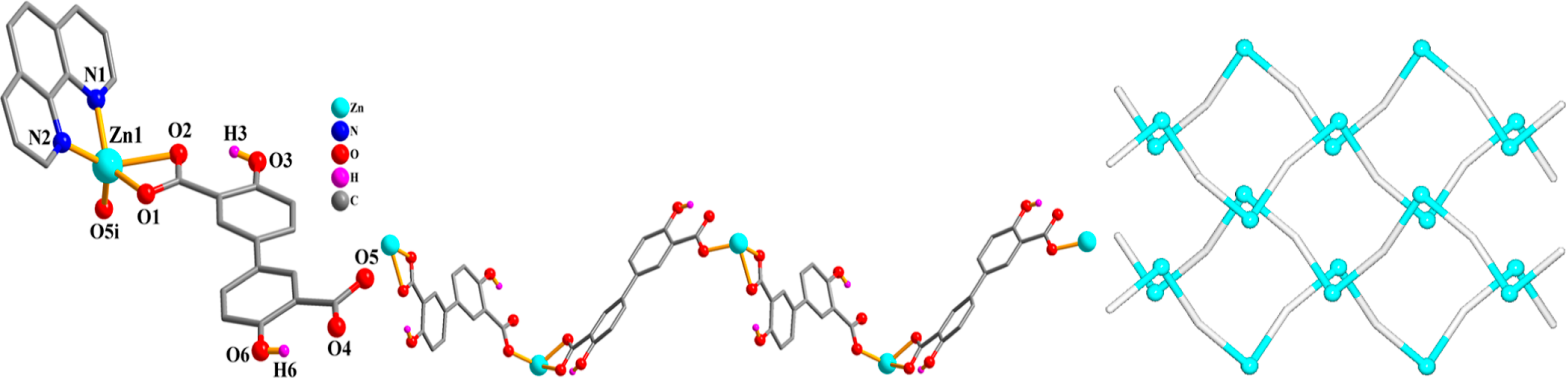
Crystal structure of **7**. (a) Connectivity and coordination environment of metal atom; CH atoms are not shown. (b) 1D zigzag chain; phen ligands are omitted; representation along the *c*-axis. (c) Topological view of four zigzag chains of the 2C1 type; centroids of μ_2_-H_2_L_2_^2–^ linkers (gray), Zn atoms (turquoise balls).

This 1D CP (Figure 7) contains a Zn(II) atom, a μ_2_-H_2_L_2_^2–^ block, and a terminal phen ligand per asymmetric entity (Figure 7a). The five-coordinate Zn1 center assumes a distorted trigonal bipyramidal {ZnN_2_O_3_} geometry that is built from three carboxylate oxygen atoms from two μ_2_-H_2_L_2_^2–^ linkers and two N_phen_ atoms. The Zn–N [2.072(5)–2.078(5) Å] and Zn–O [1.939(3)–2.329(4) Å] bonding distances agree with related literature data.^31,56,75^ The H_2_L_2_^2–^ block functions as a μ_2_-linker with mono- and bidentate carboxylate groups (Scheme 2, mode V). The carboxylate moieties are responsible for interconnection of Zn(II) atoms to produce 1D zigzag chains (Figure 7b) of a 2C1 topological type (Figure 7c).

#### [Cd(μ_3_-H_2_L_2_)(phen)]_*n*_ (**8**)

**Figure 8:**
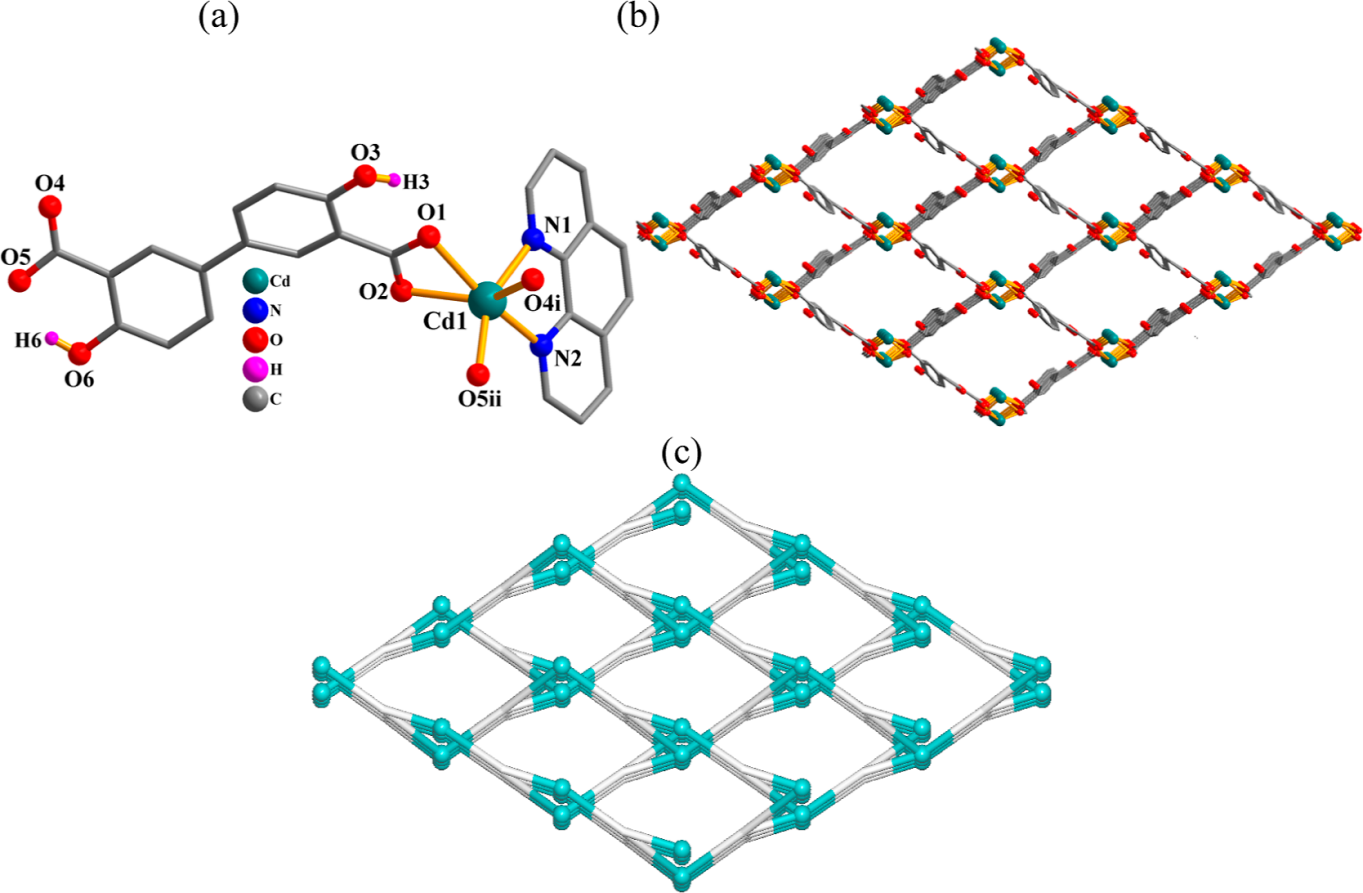
Crystal structure of **8**. (a) Connectivity and coordination environment of metal atom; CH atoms are not shown. (b) 2D metal–organic layer; phen ligands are omitted; representation along the *a*-axis. (c) Topological view of a uninodal 3-linked **utp** network; representation along the *a*-axis; centroids of 3-linked μ_2_-H_2_L_2_^2–^ nodes (gray), 3-linked Cd nodes (turquoise balls).

This 2D CP (Figure 8) comprises a Mn(II) atom, a μ_3_-H_2_L_2_^2–^ linker, and a phenanthroline ligand in the asymmetric unit. The six-coordinate Cd1 center exhibits a distorted octahedral {CdN_2_O_4_} coordination environment, which is occupied by four oxygen donors from three μ_3_-H_2_L_2_^2–^ ligands and two N_phen_ donors (Figure 8a). The bonding Cd–N [2.300(7)–2.368(7) Å] and Cd–O [2.220(6)–2.348(6) Å] distances reveal usual values.^31,39,76^ The H_2_L_2_^2–^ ligand functions as a μ_3_-linker (Scheme 2, mode VI) with bridging bidentate and bidentate carboxylate functionalities. The Cd(II) centers are united via the μ_3_-H_2_L_2_^2–^ ligands to form a two-dimensional coordination network (Figure 8b). It is assembled from the 3-linked Cd and μ_3_-H_2_L_2_^2–^ nodes that are topologically similar and give rise to a mononodal 3-connected net of a **utp** topological type; point symbol is (10^3^) (Figure 8c).^77,78^

#### [Cu(μ_2_-H_2_L_2_)(μ_2_-4,4′-bipy)(H_2_O)]_*n*_ (**9**)

**Figure 9:**
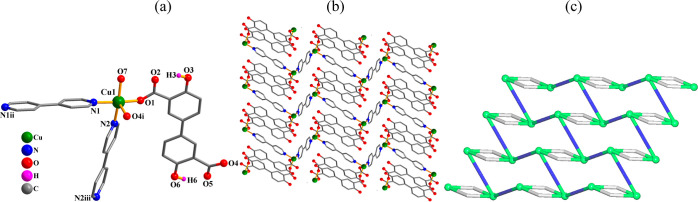
Crystal structure of **9**. (a) Connectivity and coordination environment of metal atom; CH atoms are not shown. (b) 2D CP layer; representation along the *ac* plane. (c) Topological representation of a mononodal 3-linked **hcb** net; representation along the *b*-axis; centroids of 2-connected μ_2_-H_2_L_2_^2–^ (gray) and μ_2_-4,4′**-**bipy (blue) linkers, 3-linked Cu nodes (green balls).

This CP also displays a 2D layer structure (Figure 9). The asymmetric entity encompasses a copper(II) atom, a μ_2_-H_2_L_2_^2–^ linker, a μ_2_-4,4′-bipy linker, and a water ligand. The Cu1 center is five-coordinate and discloses a distorted trigonal bipyramidal {CuN_2_O_3_} environment. This is populated by a pair of oxygen atoms from two μ_2_-H_2_L_2_^2–^ ligands, one water ligand, and two nitrogen atoms from a pair of μ_2_-4,4′-bipy linkers (Figure 9a). The Cu–N [2.000(3)–2.019(3) Å] and Cu–O [1.939(2)–2.184(3) Å] bonds agree with distances in related compounds.^20,75,79^ The H_2_L_2_^2–^ block acts as a μ-linker (Scheme 2, mode VII) with monodentate COO^–^ groups. The Cu(II) centers are held together via the μ_2_-H_2_L_2_^2–^ and μ_2_-4,4′-bipy ligands into a 2D layer (Figure 9b). Topologically, it can be defined as a mononodal 3-linked net of a **hcb** [Shubnikov hexagonal plane net/(6,3)] type (Figure 9c).^74,79^

### PXRD and TGA

For **1**–**9**, powder X-ray diffraction (PXRD) patterns were measured at ambient conditions (Figure S2, Supporting Information), revealing a good phase purity for all the compounds. This was established by comparing experimental diffraction patterns with those calculated using CIF files.

**Figure 10:**
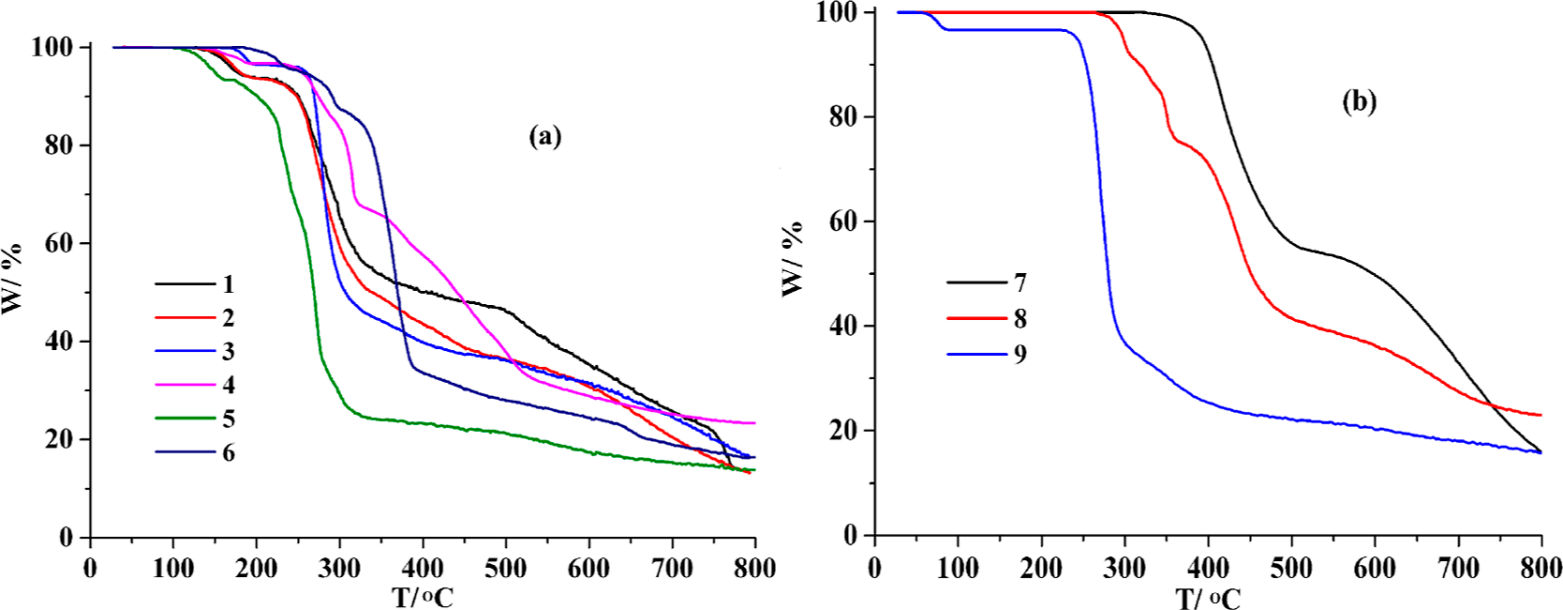
TGA traces for compounds **1**–**9** (25–800 °C, 10 °C/min, N_2_ flow).

Thermogravimetric analysis (TGA) was performed to study the thermal behavior of **1**–**9** under nitrogen flow (Figure 10). For **1**, a release of four H_2_O ligands is seen in the 126–198 °C interval (exptl, 6.4%; calcd, 6.6%), and the decomposition starts at 208 °C. Compound **2** discloses an elimination of four crystallization H_2_O molecules at 128–200 °C (exptl, 6.5%; calcd, 6.6%), pursued by degradation of the resulting solid starting from 218 °C. Compound **3** releases its water ligand between 167 and 199 °C (exptl, 3.6%; calcd, 3.5%) and the degradation of metal–organic network starts at 250 °C. Similarly, compound **4** reveals a thermal effect at 140–192 °C due to a loss of H_2_O ligand (exptl, 3.3%; calcd, 3.2%), pursued by the start of the degradation at 228 °C. In the case of CP **5**, there is a loss of two lattice H_2_O molecules (exptl, 6.7%; calcd, 6.9%) at 108–161 °C, prior to the decomposition at 174 °C. Compounds **6**–**8**, which do not contain H_2_O ligands or solvent molecules, maintain stability up to 184, 330, and 263 °C, respectively. In the case of compound **9**, a loss of water ligand (exptl, 3.4%; calcd, 3.5%) is observed in the 58–87 °C range; the resulting sample is then stable up to 227 °C.

### Catalytic Activity in Henry Reaction

**Scheme 3:**
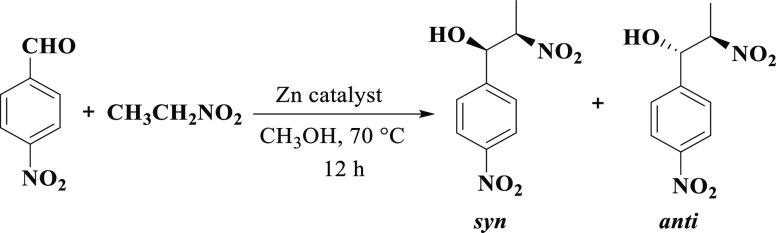
Henry Reaction of 4-Nitrobenzaldehyde (Model Substrate) with Nitroethane under Optimized Conditions

The Henry reaction, also known as nitroaldol transformation, represents an important carbon–carbon bond-forming technique in organic synthesis, wherein nitroalkanes are combined with carbonyl substrates (aldehydes, ketones) to generate beta-nitro alcohol products. Generally, this type of reaction requires a base as a catalyst, such as alkoxide, alkali metal hydroxide, or amine.^80−84^ Considering possible use of different CPs as heterogeneous catalytic systems in the Henry transformation that can proceed in the absence of base,^81−83^ the catalytic behavior of **1**–**9** was explored in the transformations involving various aldehyde substrates and nitroethane to give the corresponding β-nitro alcohol products. As a model substrate, 4-nitrobenzaldehyde was chosen (Scheme 3 and Table 3) and the impact of various reaction conditions was monitored (e.g., reaction time, type of solvent, and amount of catalyst and its recycling).

**Table 3:** Catalysis Data in the Henry Reaction of 4-Nitrobenzaldehyde and Nitroethane^a^

entry	catalyst	time (h)	amount of catalyst (mol %)	temp. (°C)	Solvent	yield (%)^b^	selectivity^c^ (syn/anti)
1	**3**	1	4	70	CH_3_OH	36	56:44
2	**3**	2	4	70	CH_3_OH	53	55:45
3	**3**	4	4	70	CH_3_OH	63	56:44
4	**3**	6	4	70	CH_3_OH	73	55:45
5	**3**	8	4	70	CH_3_OH	83	55:45
6	**3**	10	4	70	CH_3_OH	87	54:46
7	**3**	12	4	70	CH_3_OH	89	55:45
8	**3**	16	4	70	CH_3_OH	90	55:45
9	**3**	12	4	25	CH_3_OH	16	56:44
10	**3**	12	4	60	CH_3_OH	73	54:46
11	**3**	12	4	80	CH_3_OH	88	54:46
12	**3**	12	3	70	CH_3_OH	81	55:45
13	**3**	12	5	70	CH_3_OH	90	55:45
14	**3**	12	4	70	H_2_O	76	55:45
15	**3**	12	4	70	CH_3_CN	10	40:60
16	**3**	12	4	70	THF	66	44:66
17	**3**	12	4	70	C_2_H_5_OH	52	54:46
18	**1**	12	4	70	CH_3_OH	55	54:46
19	**2**	12	4	70	CH_3_OH	54	55:45
20	**4**	12	4	70	CH_3_OH	57	54:46
21	**5**	12	4	70	CH_3_OH	25	56:44
22	**6**	12	4	70	CH_3_OH	66	56:44
23	**7**	12	4	70	CH_3_OH	36	55:45
24	**8**	12	4	70	CH_3_OH	25	54:46
25	**9**	12	4	70	CH_3_OH	41	44:45
26	no catalyst	12	–	70	CH_3_OH	–	–
27	ZnCl_2_	12	4	70	CH_3_OH	12	43:57
28	2,2′-bipy	12	4	70	CH_3_OH	15	58:42
29	H_4_L_1_	12	4	70	CH_3_OH	–	–
30	H_4_L_2_	12	4	70	CH_3_OH	–	–
31	ZnCl_2_ + H_4_L_1_	12	4 + 4	70	CH_3_OH	9	42:58
32	ZnCl_2_ + H_4_L_2_	12	4 + 4	70	CH_3_OH	8	40:60

a Typical reaction conditions (unless stated otherwise): aldehyde substrate (0.5 mmol), nitroethane (2.0 mmol), catalyst (4.0 mol.%), CH_3_OH (1.0 mL), 12 h, 70 °C.

b Total product yields were calculated from the ^1^H NMR data: [moles of products per mol of substrate (aldehyde)] × 100%.

c Molar ratio between syn and anti isomers of β-nitro alcohol products.

Within the tested series of compounds **1**–**9**, the zinc(II) CP [Zn(μ_2_-H_2_L_1_)(2,2′-bipy)(H_2_O)]_*n*_ (**3**) revealed the most promising catalytic activity (Table 3) and thus was investigated in more detail. In contrast to **3**, other tested compounds showed lower efficiency with total product yields between 25 and 66% (Table 3, entries 18–25).

In the reaction catalyzed by **3**, there is an accumulation of two isomers of β-nitro alcohol products with a rise of the total yield from 36% to 90% when the reaction time was increased from 1 to 16 h (entries 1–7, Table 3; Figure S5, Supporting Information). However, the reaction is almost complete after 12 h (89% yield), and this time was used in further experiments. The catalyst amount also has an influence on the total yield, leading to its increase from 81% to 90% on varying the catalyst loading from 3 to 5 mol % (entries 7, 12, and 13). Although methanol appeared to be the solvent of choice, a number of additional solvents were screened, but these were less efficient. The following tendency in the total product yields was observed (Table 3, entries 7, 14–17): CH_3_OH (89%) > H_2_O (76%) > THF (66%) > C_2_H_5_OH (52%) > CH_3_CN (10%). Although there is no clear connection between the activity and structure of the catalyst, a superior performance of compound **3** might be related to its 1D chain structure and/or existence of open Zn sites and labile water ligands.^75,79,83,84^ Compound **3** with a 1D structure features a better accessibility of metal centers if compared to MOF **6** with a 3D structure. This likely explains superior catalytic performance of **3**.^81,82^ Besides, we have to highlight that the present Henry reactions between 4-nitrobenzaldehyde and nitroethane do not occur in the catalyst’s absence or applying organic H_4_L_1_ and H_4_L_2_ precursors as catalysts (no products were detected in all cases). Furthermore, the use of ZnCl_2_ or 2,2′-bipy as a potential catalyst revealed only low product yields of 12% and 15%, respectively (Table 3, entries 26–30). An important observation also concerns the fact that in the reactions catalyzed by **3**, there is no formation of byproducts as attested by NMR analysis (Figure S4). With regard to selectivity to syn and anti product isomers, their formation in close to equal amounts is generally observed, as expected for such type of reactions that lead to diastereomeric mixtures.^80^

Using the optimized reaction conditions, we also explored catalyst **3** for studying the substrate scope on different aldehydes (Table 4). Substituted benzaldehydes, cinnamaldehyde, and acetaldehyde were tested in the Henry reaction, leading to total product yields in the 20–92% range. In comparison to benzaldehyde (81% product yield), substituted benzaldehydes containing an electron-withdrawing functionality (e.g., −NO_2_, −Cl) revealed similar or increased product yields (81–90%; Table 4, entries 1–5); this can be potentially related to enhanced electrophilicity of these aldehydes. However, the aldehyde substrates bearing electron-donating functionalities (e.g., −OH, −CH_3_, −OCH_3_) and cinnamaldehyde displayed inferior yields of products (20–60%; Table 4, entries 6–9).

**Table 4:** Henry Reaction between Different Aldehydes and Nitroethane in the Presence of Catalyst **3**^a^

entry	benzaldehyde (RC_6_H_4_CHO) or other aldehyde substrate	product yield (%)^b^	selectivity (syn/anti)^c^
1	R = H	81	51:49
2	R = 2-NO_2_	82	58:42
3	R = 3-NO_2_	85	55:45
4	R = 4-NO_2_	89	55:45
5	R = 4-Cl	81	50:50
6	R = 4-OH	60	56:44
7	R = 4-CH_3_	56	43:57
8	R = 4-OCH_3_	20	45:54
9	cinnamaldehyde	56	57:43
10	Acetaldehyde	92	56:44

a Reaction parameters: aldehyde substrate (0.5 mmol), nitroethane (2.0 mmol), CP **3** (4.0 mol %), CH_3_OH (1.0 mL), 12 h, 70 °C.

b Yields were calculated from the ^1^H NMR data: [moles of products per mol of substrate (aldehyde)] × 100%.

c Molar ratio between syn and anti isomers of β-nitro alcohol products.

The stability of catalyst **3** and its performance after recycling were also evaluated (conditions of Table 3, entry 7). The results of these catalytic experiments along with PXRD data (Figures S6 and S7, Supporting Information) point out that compound **3** preserves its structure and features, resembling catalytic performance during five reaction cycles. This can be evidenced by almost constant yields of products, a minor decline of which is likely associated with a minor loss of catalyst after several recycling experiments. In a model reaction involving 4-nitrobenzaldehyde and nitroethane as substrates, the catalytic performance of **3** is comparable to other heterogeneous catalytic systems based on metal-carboxylate coordination compounds (Table S3, Supporting Information)^81−86^ or is superior if the reaction time is taken into consideration. Apart from high activity, good stability, and recyclability of **3**, this catalyst can lead to excellent product yields in a shorter reaction time.

For these Henry reactions, a possible mechanism was proposed on the basis of prior research studies describing related transformations that involve aldehydes and nitroethane and are catalyzed by CPs (Figure S8, Supporting Information).^87−89^ Hence, there is an initial activation of both 4-nitrobenzaldehyde and nitroethane via an interaction with Zn(II) sites of the CP (Figure S8, step i). Such an interaction augments electrophilicity of 4-nitrobenzaldehyde and acidity of nitroethane. Subsequently, a reactive nitronate species is produced via deprotonation of the activated acidic nitroethane (step ii). Then, the new C–C bond is formed by nucleophilic attack of nitronate ion to coordinated 4-nitrobenzaldehyde (step iii). Finally, the next molecule of 4-nitrobenzaldehyde binds to the zinc(II) center, resulting in the release of the corresponding β-nitro alcohol product and completion of the catalytic cycle (Figure S8, step iv).

## Conclusions

In the present work, the use of 3,3′-dihydroxy-(1,1′-biphenyl)-4,4′-dicarboxylic (H_4_L_1_) and 4,4′-dihydroxy-(1,1′-biphenyl)-3,3′-dicarboxylic (H_4_L_2_) acids was further explored in the hydrothermal generation of nine new coordination compounds **1**–**9**. All the obtained products were completely characterized, and their structures and topologies were established. Remarkably, crystal structures of MOFs **5** and **6** disclosed two- or fivefold 3D + 3D interpenetrated nets, respectively, thus contributing to broadening an important family of interpenetrated metal–organic architectures.^90,91^

Apart from standard investigation of thermal stability and luminescence characteristics, the obtained products were screened as catalysts in the Henry reaction between aldehydes and nitroethane to give β-nitro alcohol products. A zinc(II) 1D CP **3** revealed a particularly notable catalytic behavior that was optimized to a variety of reaction parameters and substrate scope, thus leading to up to 90% total product yields. Furthermore, this catalyst exhibited good stability and the possibility of reuse for five cycles.

In summary, the obtained compounds represent novel examples of metal–organic architectures that were hydrothermally assembled from hydroxy-functionalized biphenyl dicarboxylate blocks and different types of crystallization mediators. These results will stimulate further use of H_4_L_1_, H_4_L_2_ and related dicarboxylate linkers for generating functional CPs and MOFs.

## Supporting Information Available

Full synthetic procedures and analytical data for **1**–**9**, FTIR spectra, PXRD patterns, luminescence data, catalysis data, and bonding parameters (PDF)
