# Supplementary material for: Coordination Polymers from Biphenyl-Dicarboxylate Linkers: Synthesis, Structural Diversity, Interpenetration, and Catalytic Properties
Source: Inorg Chem. 2022 Aug 3;61(32):12577–90. doi: 10.1021/acs.inorgchem.2c01488 (PMC9775469; doi:10.1021/acs.inorgchem.2c01488)
Supplement: Supplementary file 1 — ic2c01488_si_001.pdf [file ic2c01488_si_001.pdf]

Supporting Information (SI)

Coordination Polymers from Biphenyl-Dicarboxylate Linkers: Synthesis, Structural Diversity, Interpenetration, and Catalytic Properties

Xiaoyan Cheng,[†] Lirong Guo,*[,†] Hongyu Wang,[†] Jinzhong Gu,*[,†] Ying Yang,[†] Marina V. Kirillova,[‡] and Alexander M. Kirillov*[,‡]

[†]*State Key Laboratory of Applied Organic Chemistry, Key Laboratory of Nonferrous Metal Chemistry and Resources Utilization of Gansu Province, College of Chemistry and Chemical Engineering, Lanzhou University, Lanzhou 730000, People's Republic of China*

[‡]*Centro de Química Estrutural, Institute of Molecular Sciences, Departamento de Engenharia Química, Instituto Superior Técnico, Universidade de Lisboa, Av. Rovisco Pais, 1049-001, Lisbon, Portugal*

*To whom correspondence should be addressed. Tel.: +86-931-8915196; E-mail: gujzh@lzu.edu.cn; guolr@lzu.edu.cn; kirillov@tecnico.ulisboa.pt; Tel.: +351-218419396.

Contents

	General methods	p. S2
	Synthesis and analytical data for **1–9**	p. S2
**Figure S1**	Transmittance FTIR spectra of compounds **1–9**	p. S5
**FigureS2**	PXRD patterns of compounds **1–9**	p. S7
	Luminescent properties of compounds **1–9**	p. S7
**Figure S3**	Solid-state emission spectra of **1–9**, H_4L_1 and H_4L_2	p. S8
**Figure S4**	Typical ^1H NMR spectrum of the reaction mixture	p. S8
	Calculation of the product yield and selectivity	p. S8
**Figure S5**	Accumulation of products vs. time	p. S9
**Figure S6**	Catalyst recycling experiments	p. S9
**Figure S7**	PXRD patterns for **3**	p. S10
**Figure S8**	Proposed catalytic cycle for Henry reaction catalyzed by **3**	p. S11
**Table S1**	Selected bond lengths and angles for compounds **1–9**	p. S10
**Table S2**	Hydrogen bonds in crystal packing of **1–9**	p. S12
**Table S3**	Comparison of catalytic systems	p. S14
	Supporting references	p. S14

General Methods. All chemicalsand solvents were obtained from commercial suppliers. 3,3′-Dihydroxy-(1,1′-biphenyl)-4,4′-dicarboxylic acid (H_4L_1) and 4,4′-dihydroxy-(1,1′-biphenyl)-3,3′-dicarboxylic acid (H_4L_2) were acquired from Jinan Henghua Sci. & Tec. Co., Ltd.C/N/H analyses were run on an Elementar Vario EL elemental analyzer. Bruker EQUINOX 55 spectrometer was used for recording the FTIR spectra (KBr discs). LINSEIS STA PT1600 thermal analyzer was used for thermogravimetric (TGA) measurements (heating rate: 10°C/min; N_2 flow). PXRD (powder X-ray diffraction) analyses were carried out on a Rigaku-Dmax 2400 diffractometer (Cu-Kα radiation, λ= 1.54060 Å). Solid-state excitation and emission spectra were measured on an Edinburgh FLS920 fluorescence spectrometer under ambient temperature. Solution ^{1}H NMR spectra were recorded on a JNM ECS 400M spectrometer.

Synthesis and analytical data for 1–9.

*$[Co_2(\mu_2\text{-}H_2L_1)_2(phen)_2(H_2O)_4]$ (**1**).* A mixture of $CoCl_2·6H_2O$ (0.2 mmol, 47.6 mg), H_4L_1(0.2 mmol, 54.8 mg), phen (0.2 mmol, 40.0 mg), and NaOH (0.4 mmol, 16.0 mg) in H_2O (10 mL) was stirred for 15 min at ambient temperature. It was then sealed in a Teflon-lined stainless steel reactor (25 mL) and heated at 160 °C for 3 days, followed by a slow cooling to ambient temperature (10 °C/h). Pink block-shaped crystals were isolated manually, washed with distilled water, and dried in air to give product **1**. Yield: 54% (based on H_4L_1). Calcd for $C_{52}H_{40}Co_2N_4O_{16}$: C 57.05, H 3.68, N 5.12%. Found: C 57.29, H 3.66, N 5.10%. IR (KBr, cm^{-1}): 3435w, 3076 w, 1625 m, 1577 s, 1512 m, 1485 w, 1425s, 1349 m, 1225 w, 1189 w, 1157 w, 1141 w, 1101 w, 1033 w, 962 w, 865 m, 850 w, 810 w, 726 m, 670 w, 641 w.

*$[Mn(\mu_4\text{-}H_2L_1)(phen)]_n·4nH_2O$ (**2**).* A mixture of $MnCl_2·4H_2O$ (39.6 mg, 0.2 mmol), H_4L_1 (0.2 mmol, 54.8 mg), phen (40.0 mg, 0.2 mmol), NaOH (16.0 mg, 0.4 mmol), and H_2O (10 mL) was stirred at room temperature for 15 min, then sealed in a 25 mL Teflon-lined stainless steel vessel, and heated at 160 °C for 3 days, followed by cooling to room temperature at a rate of 10 °C·h^{-1}. Yellow crystals of **2** were isolated manually, and washed with distilled water. Yield: 46% (based on H_4L_1). Anal. Calcd for $C_{52}H_{40}Mn_2N_4O_{16}$: C 57.87, H 3.71, N 5.16%. Found: C 57.58, H 3.72, N 5.15%. IR (KBr, cm^{-1}): 3067 w, 1625 m, 1580 s, 1517 m, 1445 s, 1369 s, 1313 w, 1233 w, 1185 w, 1154 w, 1101 w, 1045 w, 962 w, 869 m, 845 w, 814 w, 786 w, 726 m, 705 w, 665 w, 637 w.

*$[Zn(\mu_2\text{-}H_2L_1)(2,2'\text{-}bipy)(H_2O)]_n$ (**3**).* A mixture of $ZnCl_2$ (27.3 mg, 0.20 mmol), H_4L_1 (0.2 mmol, 54.8 mg), 2,2′-bipy (31.2 mg, 0.2 mmol), NaOH (16.0 mg, 0.4 mmol), and H_2O (10 mL) was stirred at room temperature for 15

min, then sealed in a 25 mL Teflon-lined stainless steel vessel, and heated at 160 °C for 3 days, followed by cooling to room temperature at a rate of 10 °C·h^{-1}. Colorless block-shaped crystals of **3** were isolated manually, washed with distilled water and dried (yield 51% based on H_4L_1). Anal. Calcd for $C_{24}H_{18}ZnN_2O_7$: C, 56.32; H, 3.54; N, 5.47. Found: C, 56.47; H, 3.52; N, 5.44%. IR (KBr, cm^{-1}): 3435 w, 3043 w, 1632 m, 1580 s, 1513 w, 1489 m, 1433 s, 1357 m, 1333 m, 1249 w, 1229 m, 1185 w, 1157 w, 1105 w, 1057 w, 1026 w, 961 w, 869 m, 817 w, 769 m, 729 w, 709 w, 670 w, 629 w.

[Cd(μ_2-H_2L_1)(2,2'-bipy)(H_2O)]$_n$ (4). A mixture of $CdCl_2·H_2O$ (40.2 mg, 0.20 mmol), H_4L_1 (0.2 mmol, 54.8 mg), bipy (31.2 mg, 0.2 mmol), NaOH (16.0 mg, 0.4 mmol), and H_2O (10 mL) was stirred at room temperature for 15 min, then sealed in a 25 mL Teflon-lined stainless steel vessel, and heated at 160 °C for 3 days, followed by cooling to room temperature at a rate of 10 °C·h^{-1}. Colorless block-shaped crystals of **4** were isolated manually, washed with distilled water and dried (yield 46% based on H_4L_1). Anal. Calcd for $C_{24}H_{18}CdN_2O_7$: C, 51.58; H, 3.25; N, 5.01. Found: C, 51.39; H, 3.28; N, 5.03%. IR (KBr, cm^{-1}): 3416 w, 3060 w, 1625 m, 1585 s, 1517 m, 1429 s, 1385 s, 1225 w, 1141 w, 1105 w, 1045 w, 905 w, 850 m, 773 w, 726 m, 665 w, 641 w.

*[Mn_2(μ_2-H_2L_1)(μ_4-H_2L_1)(μ_2-4,4'-bipy)$_2$]$_n$·4nH_2O (**5**).* A mixture of $MnCl_2·4H_2O$ (39.6 mg, 0.2 mmol), H_4L_1 (0.2 mmol, 54.8 mg), 4,4′-bipy (31.2 mg, 0.2 mmol), NaOH (16.0 mg, 0.4 mmol), and H_2O (10 mL) was stirred at room temperature for 15 min, then sealed in a 25 mL Teflon-lined stainless steel vessel, and heated at 160 °C for 3 days, followed by cooling to room temperature at a rate of 10 °C·h^{-1}. Yellow block-shaped crystals of **5** were isolated manually, washed with distilled water and dried (yield 44% based on H_4L_1). Anal. Calcd for $C_{48}H_{38}Mn_2N_4O_{16}$: C, 55.61; H, 3.69; N, 5.40. Found: C, 55.74; H, 4.67; N, 5.38%. IR (KBr, cm^{-1}): 3143 m, 1605 m, 1577 s, 1516 w, 1493 w,1425 m, 1353 s, 1249 w, 1154 w, 1069 w, 1029 w, 1002 w, 957 w, 857 m, 805 m, 777 w, 726 w, 701 w, 674 w, 650 w.

[Zn(μ_2-H_2L_1)(μ_2-4,4'-bipy)]$_n$ (6). Synthesis of **6** was similar to **5** except using $ZnCl_2$ (27.3 mg, 0.20 mmol) instead of $MnCl_2·4H_2O$. Colorless block-shaped crystals of **6** were isolated manually, washed with distilled water and dried (yield 43% based on H_4L_1). Anal. Calcd for $C_{24}H_{16}ZnN_2O_6$: C, 58.37; H, 3.27; N, 5.67. Found: C, 58.53; H, 3.25; N, 5.68%. IR (KBr, cm^{-1}): 1616 s, 1577 s, 1489 m,1421 s, 1389 m, 1333 s, 1229 m, 1157 w, 1069 w, 1029 w, 1014 w, 962 w, 865 m, 814 m, 729 w, 705 w, 673 w, 641 w.

[Zn(μ_2-H_2L_2)(phen)]$_n$ (7). A mixture of $ZnCl_2$ (27.3 mg, 0.20 mmol), H_4L_2 (54.8 mg, 0.20 mmol), phen (40.0 mg, 0.20 mmol), NaOH (16.0 mg, 0.40 mmol), and H_2O (10 mL) was stirred at room temperature for 15 min, then sealed in a 25 mL Teflon-lined stainless steel vessel, and heated at 160 °C for 3 days, followed by cooling to room temperature at a rate of 10 °C·h^{-1}. Colorless block-shaped crystals of **7** were isolated manually, washed with distilled water and dried (yield 53% based on H_4L_2). Anal. Calcd for $C_{26}H_{16}ZnN_2O_6$: C, 60.31; H, 3.11; N, 5.41.

Found: C, 60.53; H, 3.13; N, 5.38%.IR (KBr, cm^{-1}): 1630 w, 1556 s, 1470 s, 1415 s, 1362 w, 1288 w, 1242 m, 1146 w, 1106 w, 1047 w, 981 w, 936 w, 874 m, 836 w, 774 w, 720 w, 698 w, 641 w.

*$[Cd(\mu_3\text{-}H_2L_2)(phen)]_n$ (**8**).* A mixture of $CdCl_2{\cdot}H_2O$ (40.2 mg, 0.2 mmol), H_4L_2 (54.8 mg, 0.20 mmol), phen (40.0 mg, 0.20 mmol), NaOH (16.0 mg, 0.40 mmol), and H_2O (10 mL) was stirred at room temperature for 15 min, then sealed in a 25 mL Teflon-lined stainless steel vessel, and heated at 160 °C for 3 days, followed by cooling to room temperature at a rate of 10 °C·h^{-1}. Colorless block-shaped crystals of **8** were isolated manually, washed with distilled water and dried (yield 45% based on H_4L_2). Anal. Calcd for $C_{26}H_{16}CdN_2O_6$: C, 55.29; H, 2.86; N, 4.96. Found: C, 55.16; H, 2.84; N, 4.97%.IR (KBr, cm^{-1}): 1626 w, 1560 s, 1514 w, 1481 w, 1419 s, 1374 w, 1324 w, 1282 w, 1241 w, 1220 w, 1196 w, 1162 w, 1146 w, 1096 w, 1043 w, 997 w, 914 w, 868 w, 831 s, 724 m, 699 w, 641 w.

*$[Cu(\mu_2\text{-}H_2L_2)(\mu_2\text{-}4,4'\text{-bipy})(H_2O)]_n$ (**9**).* A mixture of $CuCl_2{\cdot}2H_2O$ (34.1 mg, 0.2 mmol), H_4L_2 (54.8 mg, 0.20 mmol), 4,4′-bipy (31.2 mg, 0.20 mmol), NaOH (24.0 mg, 0.60 mmol), and H_2O (10 mL) was stirred at room temperature for 15 min, then sealed in a 25 mL Teflon-lined stainless steel vessel, and heated at 160 °C for 3 days, followed by cooling to room temperature at a rate of 10 °C·h^{-1}. Green needle-shaped crystals of **9** were isolated manually, washed with distilled water and dried (yield 43% based on H_4L_2). Anal. Calcd for $C_{24}H_{17}CuN_2O_7$: C, 56.64; H, 3.37; N, 5.50. Found: C, 56.75; H, 3.40; N, 5.48%.IR (KBr, cm^{-1}): 3434 w, 3100 w, 1627 w, 1564 s, 1471 m, 1407 s, 1358 w, 1286 w, 1246 w, 1157 w, 1069 w, 944 w, 872 m, 852 w, 820 m, 727 w, 695 w, 647 w.

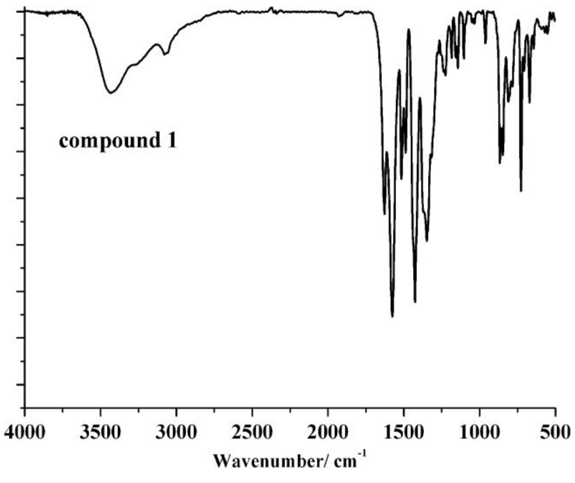

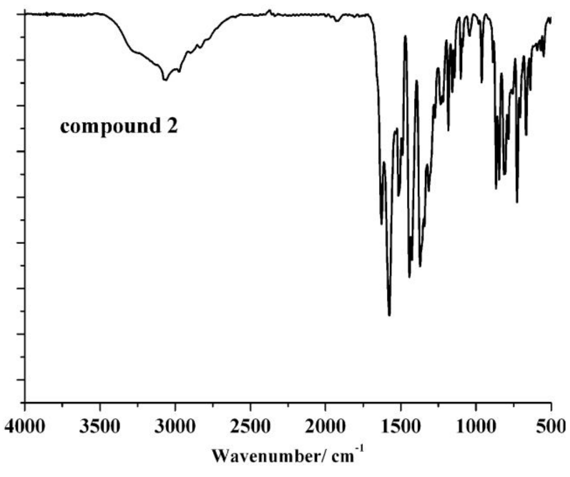

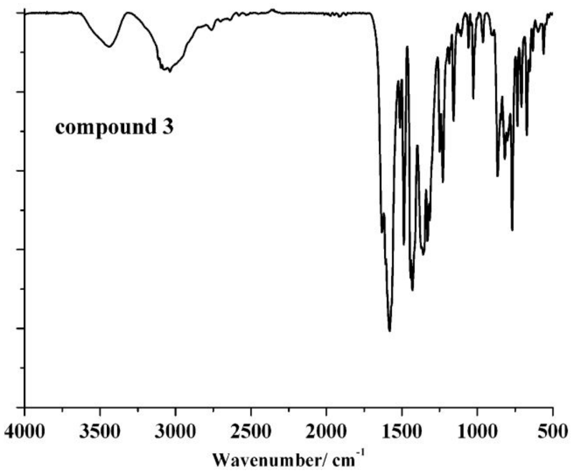
compound 3
4000 3500 3000 2500 2000 1500 1000 500
Wavenumber/ cm-1

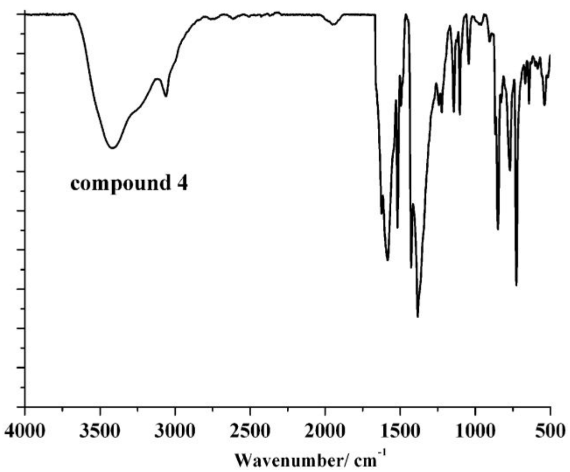
compound 4
4000 3500 3000 2500 2000 1500 1000 500
Wavenumber/ cm-1

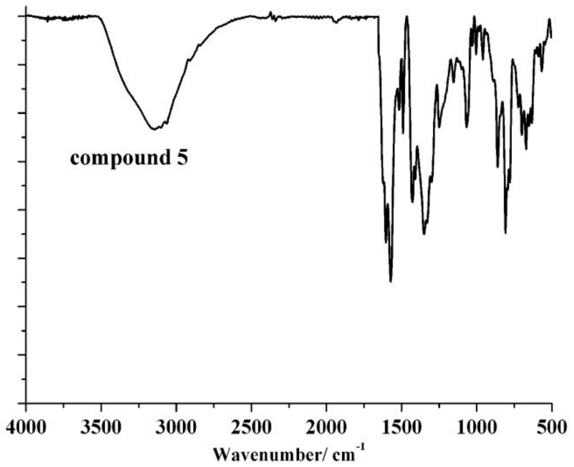
compound 5
4000 3500 3000 2500 2000 1500 1000 500
Wavenumber/ cm-1

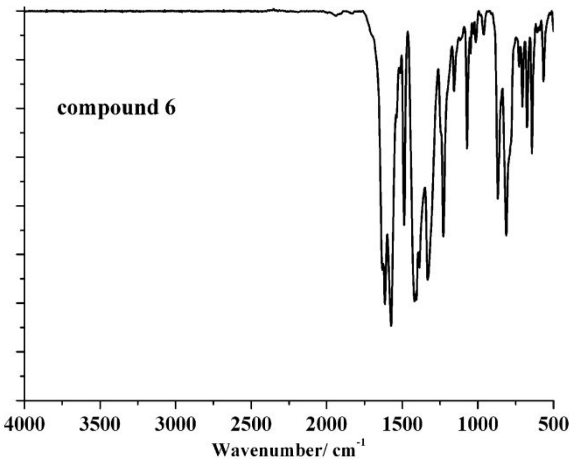
compound 6
4000 3500 3000 2500 2000 1500 1000 500
Wavenumber/ cm-1

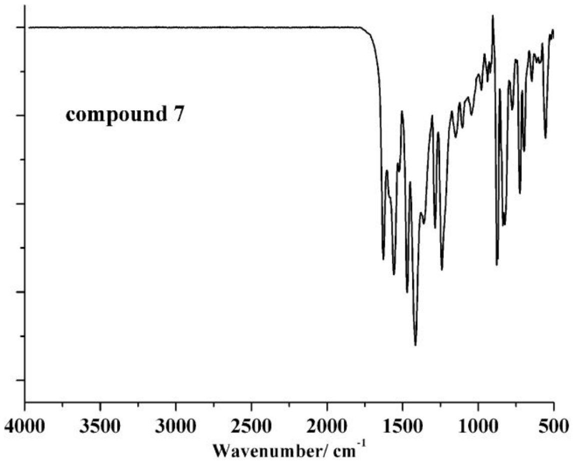
compound 7
4000 3500 3000 2500 2000 1500 1000 500
Wavenumber/ cm-1

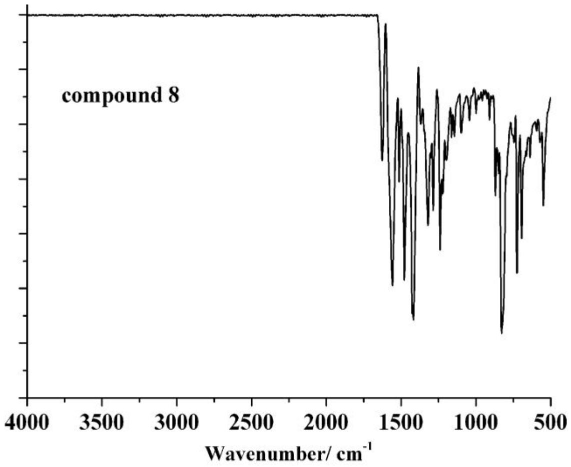
compound 8
4000 3500 3000 2500 2000 1500 1000 500
Wavenumber/ cm-1

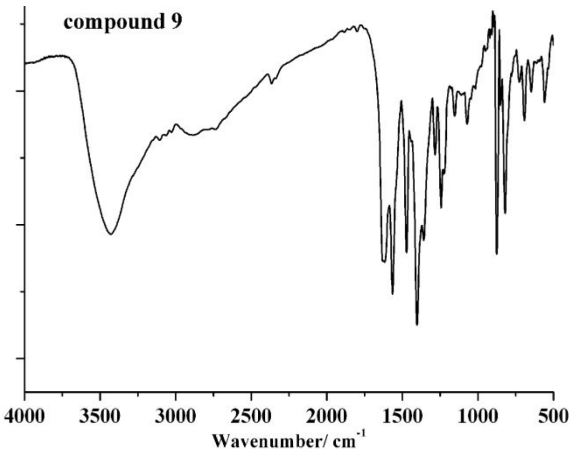

Figure S1. Transmittance (%) FTIR spectra of compounds **1–9**.

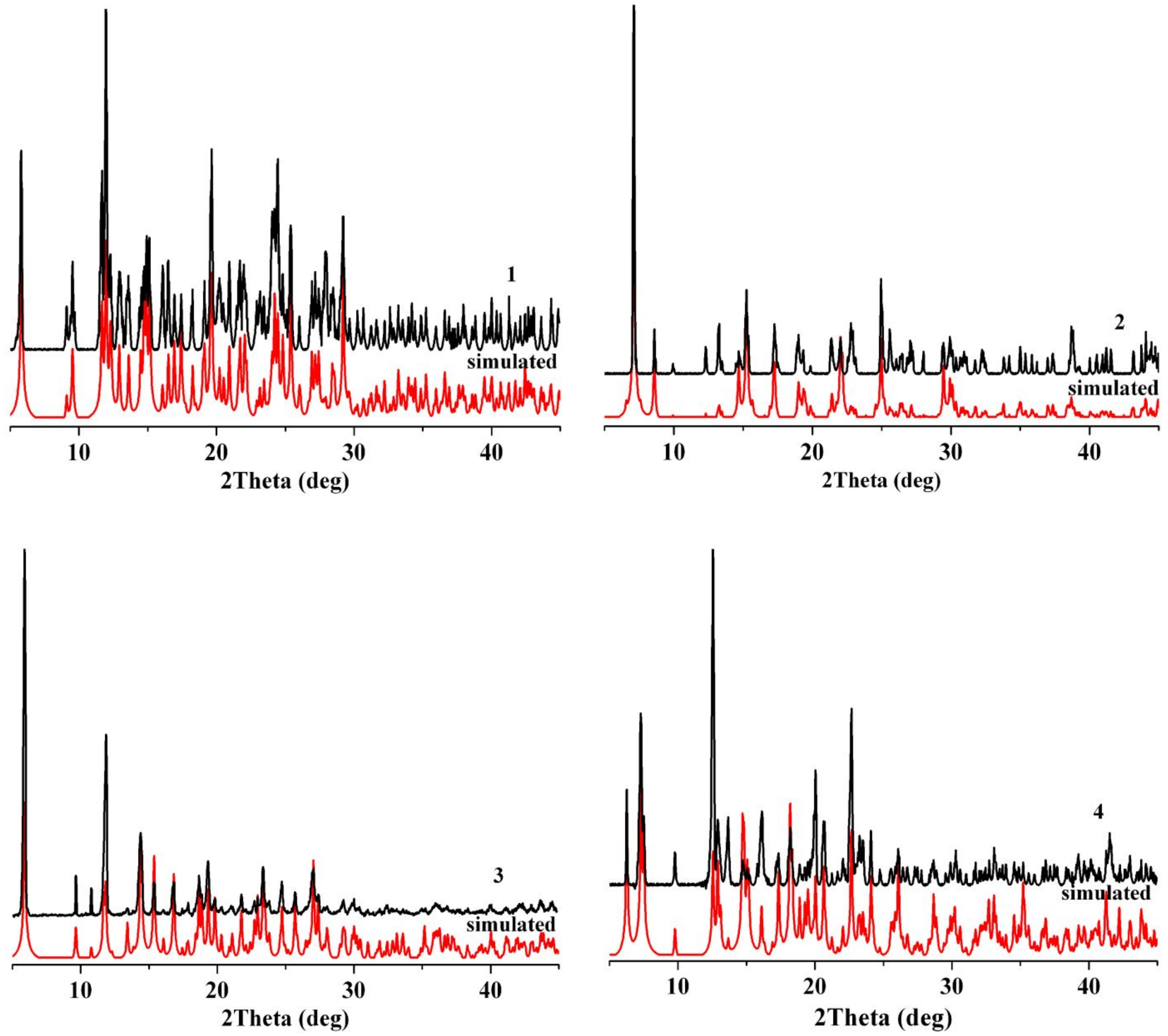

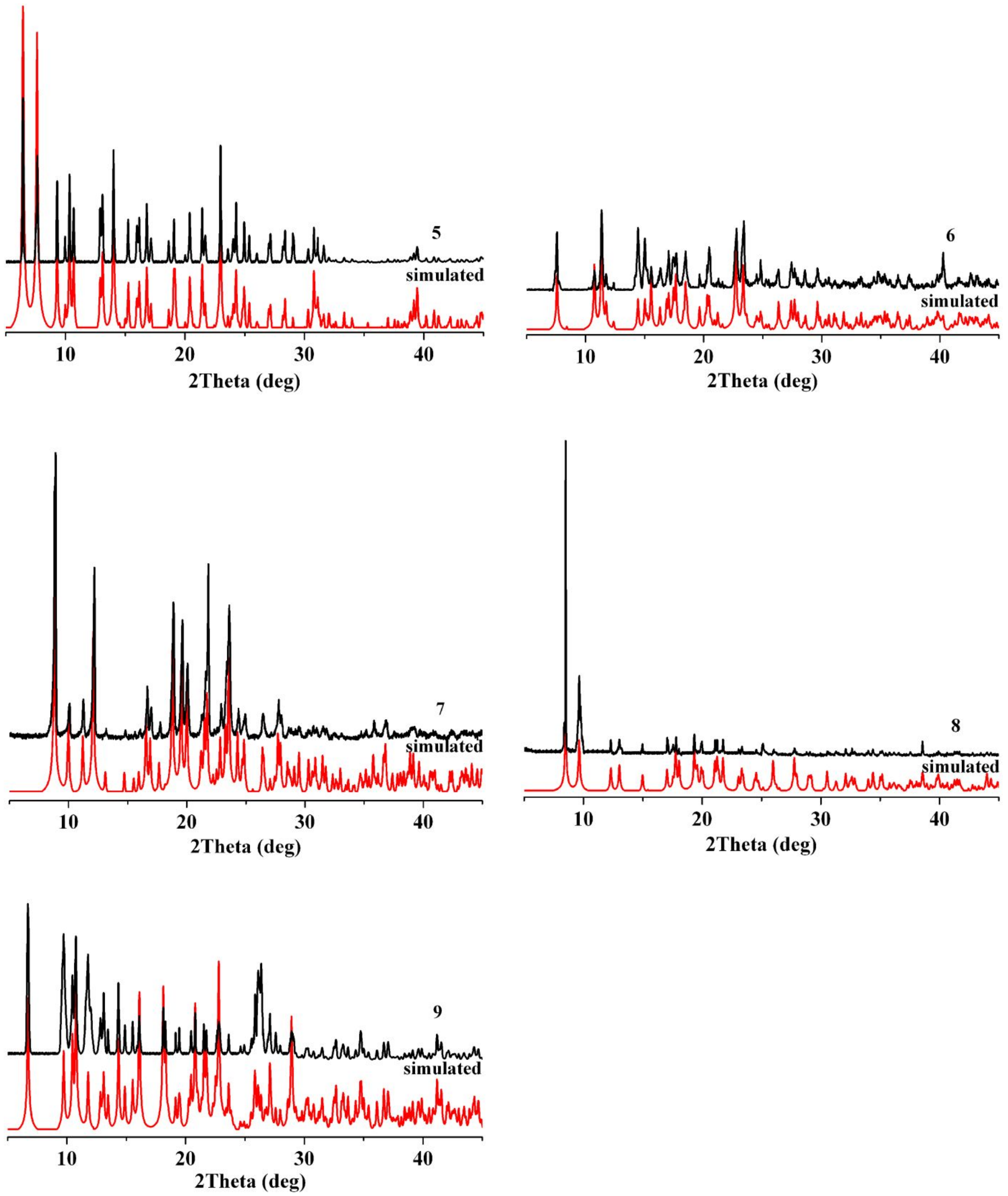

Figure S2. PXRD patterns of compounds **1–9** at room temperature. Black patterns correspond to the experimental data obtained using the as-synthesized bulk samples. Red patterns were simulated from the single crystal X-ray data (CIF files).

Luminescent Properties. The emission spectra of compounds **1–9**, H_4L_1 and H_4L_2 were recorded in the solid state

at room temperature (Fig. S3). The spectra of H_4L_1 and H_4L_2 disclose two weak emission bands centered at 470 and 483 nm. In contrast to H_4L_1 and H_4L_2, zinc(II) and cadmium(II) derivatives feature bands of a more pronounced intencity with maxima in the 440−458 nm range, namely 457 nm for **3**, 440 nm for **4**, 458 nm for **6**, 455 nm for **7**, and 445 nm for **8**. These bands are associated with an intraligand π–π* or n–π* transitions of main carboxylate ligand.[S1–S3] An enhanced luminescence of **3**, **4**, and **6**–**8** vs. H_4L_1 and H_4L_2 is likely due to the coordination of ligands to Zn(II) or Cd(II), which may strengthen the rigidity of ligands and diminish a loss of energy from radiationless decay.[S3–S5] However, compounds **1**, **2**, **5**, and **9** display very weak luminescence and almost no emission, which is probably attributed to the fluorescence quenching of Co^{2+}, Mn^{2+}, and Cu^{2+} by the ligands present in these compounds.[S6–S9]

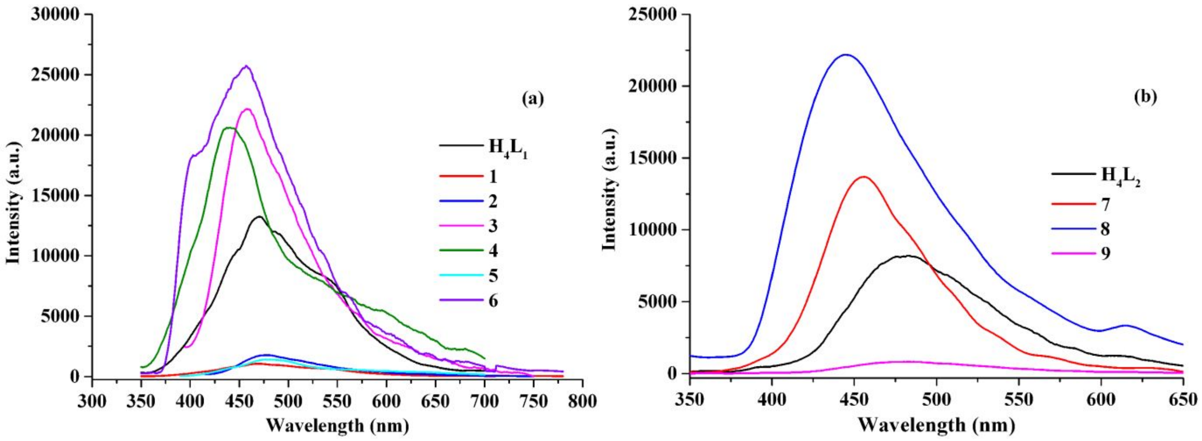

Figure S3. Solid-state emission spectra of **1**–**9**, H_4L_1 and H_4L_2 at room temperature (λ_{ex}= 316 nm).

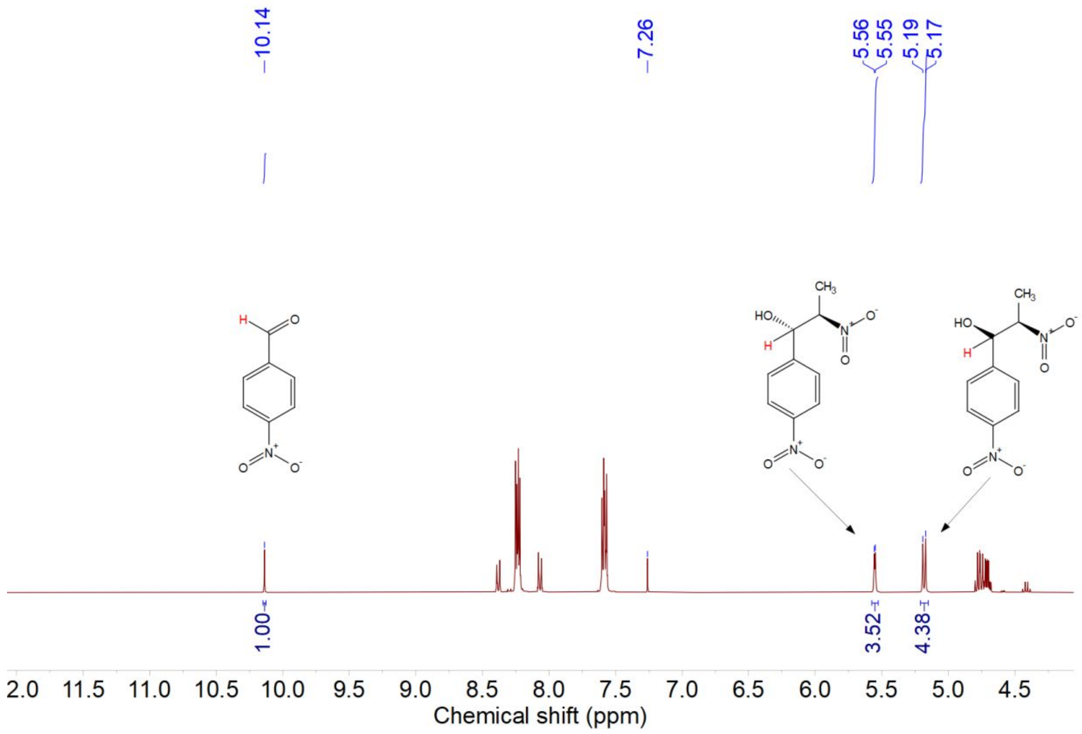

Figure S4. Typical ^{1}H NMR spectrum of the reaction mixture with integration of signals for determination of the Henry reaction products (conditions of Table 3, entry 7; 4-nitrobenzaldehyde substrate, catalyst **3**).

Calculation of the product yield and selectivity based on the data of Figure S4

Yield:

Total amount of compounds: 4-nitrobenzaldehyde + *anti* + *syn* = 1.00 + 3.52 + 4.38 = 8.90.

Percentage of the unreacted 4-nitrobenzaldehyde: (1/8.90) × 100 = 11.2%.

Conversion of 4-nitrobenzaldehyde = yield of beta-nitroalkanols = 100−11.2= 88.8%.

Selectivity:

Selectivity toward *anti* product: 3.52/(3.52 + 4.38) × 100 = 45%.

Selectivity toward *syn* product: 4.38/(3.52 + 4.38) × 100 = 55%.

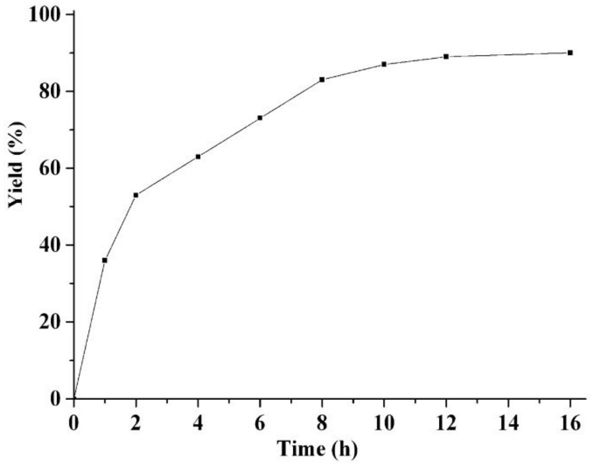

Figure S5. Accumulation of product vs. time in the Henry reaction of 4-nitrobenzaldehyde with nitroethane catalysed by **3**. Reaction conditions are those of Table 3, entries 1−7.

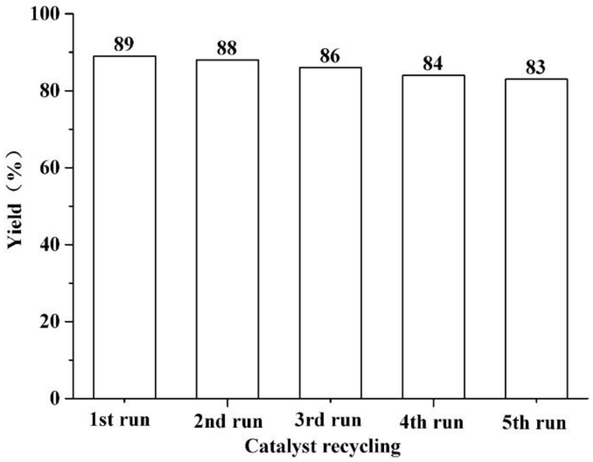

Figure S6. Catalyst recycling experiments (five reaction runs) in the Henry reaction of 4-nitrobenzaldehyde with nitroethane catalyzed by **3**. Reaction conditions are those of Table 3, entry 7. Figures above the bars correspond to product yields in %.

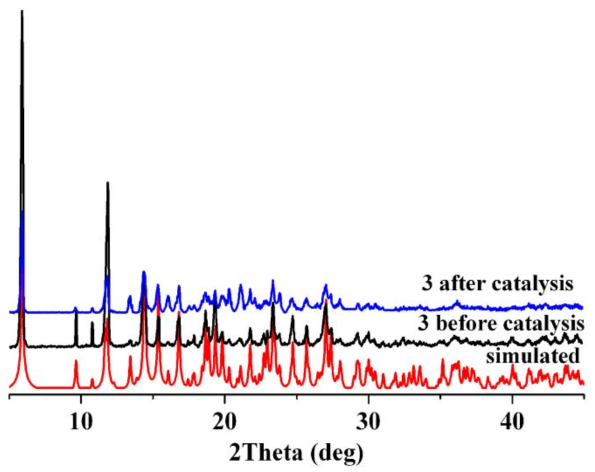

Figure S7. PXRD patterns for **3**: simulated (red), before (black) and after (blue) catalysis.

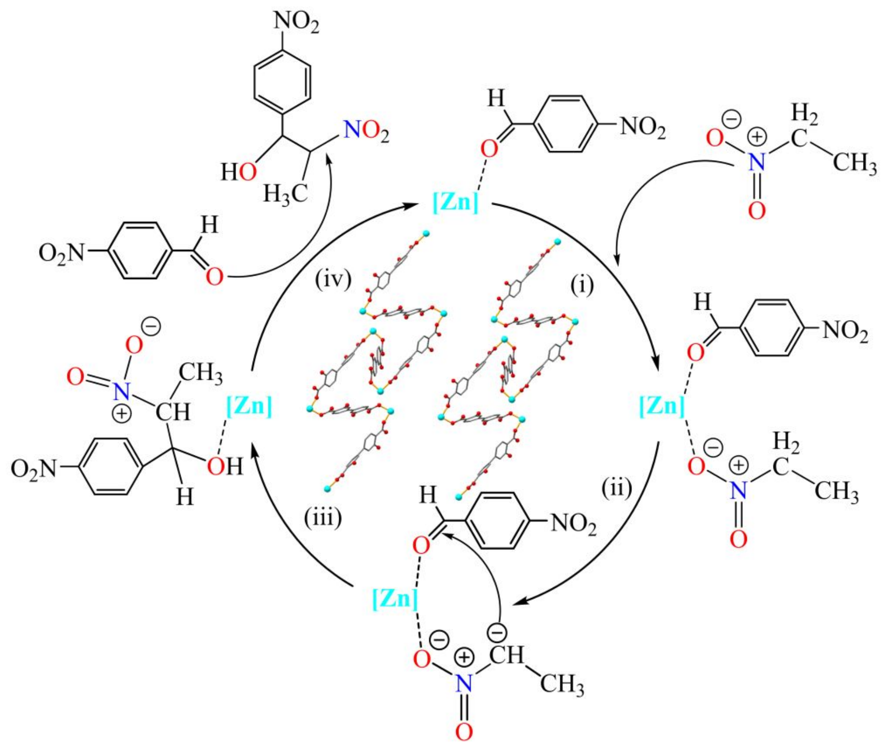

Figure S8. Proposed catalytic cycle for Henry reaction catalyzed by **3**.

Table S1. Selected bond lengths [Å] and angles [°] for compounds **1–9**.[a]

**1**					
Co(1)-O(1)	2.0418(12)	Co(1)-O(5)i	2.1838(13)	Co(1)-O(7)	2.0866(13)
Co(1)-O(8)	2.1384(14)	Co(1)-N(1)	2.1319(16)	Co(1)-N(2)	2.1225(15)
O(1)-Co(1)-O(7)	95.42(6)	O(1)-Co(1)-N(2)	170.89(6)	N(2)-Co(1)-O(7)	93.64(6)
O(1)-Co(1)-N(1)	93.01(6)	O(7)-Co(1)-N(1)	169.66(6)	N(2)-Co(1)-N(1)	78.08(6)
O(8)-Co(1)-O(1)	87.43(6)	O(7)-Co(1)-O(8)	86.62(6)	N(2)-Co(1)-O(8)	92.19(6)
N(1)-Co(1)-O(8)	99.76(6)	O(5)i-Co(1)-O(1)	82.53(5)	O(5)i-Co(1)-O(7)	87.00(5)
N(2)-Co(1)-O(5)i	98.88(5)	N(1)-Co(1)-O(5)i	88.16(6)	O(5)i-Co(1)-O(8)	167.55(5)
**2**					
Mn(1)-O(1)	2.0870(16)	Mn(1)-O(2)i	2.2087(13)	Mn(1)-O(7)	2.1005(15)
Mn(1)-O(10)ii	2.2014(13)	Mn(1)-N(1)	2.3077(17)	Mn(1)-N(2)	2.3044(17)
Mn(2)-O(4)iii	2.1973(14)	Mn(2)-O(5)iv	2.0966(17)	Mn(2)-O(8)v	2.1959(14)
Mn(2)-O(11)	2.0919(16)	Mn(2)-N(3)	2.3011(18)	Mn(2)-N(4)	2.3043(19)
O(1)- Mn(1)-O(7)	108.37(8)	O(1)-Mn(1)-O(10)ii	87.78(6)	O(7)-Mn(1)-O(10)ii	99.39(6)
O(1)- Mn(1)-O(2)i	97.98(6)	O(7)-Mn(1)-O(2)i	88.63(6)	O(10)ii-Mn(1)-O(2)i	168.18(5)
O(1)- Mn(1)-N(2)	163.05(7)	N(2)-Mn(1)-O(7)	88.56(7)	N(2)-Mn(1)-O(10)ii	88.57(6)
N(2)-Mn(1)-O(2)i	82.92(6)	O(1)-Mn(1)-N(1)	90.96(7)	N(1)-Mn(1)-O(7)	160.65(7)
N(1)-Mn(1)-O(10)ii	80.00(6)	N(1)-Mn(1)-O(2)i	89.55(6)	N(1)-Mn(1)-N(2)	72.10(6)
O(11)-Mn(2)-O(5)iv	102.29(8)	O(11)-Mn(2)-O(8)v	97.62(6)	O(8)v-Mn(2)-O(5)iv	90.12(7)
O(11)-Mn(2)-O(4)iii	86.97(6)	O(4)iii-Mn(2)-O(5)iv	100.64(6)	O(4)iii-Mn(2)-O(8)v	167.18(6)
O(11)-Mn(2)-N(3)	164.20(8)	O(5)iv-Mn(2)-N(3)	93.51(8)	O(8)v-Mn(2)-N(3)	82.10(6)
O(4)iii-Mn(2)-N(3)	90.23(6)	O(11)-Mn(2)-N(4)	91.86(7)	O(5)iv-Mn(2)-N(4)	165.66(7)
O(8)v-Mn(2)-N(4)	85.64(6)	O(4)iii-Mn(2)-N(4)	82.24(6)	N(3)-Mn(2)-N(4)	72.35(7)
**3**					
Zn(1)-O(1)	1.9897(17)	Zn(1)-O(4)	2.0413(15)	Zn(1)-O(7)	2.0096(16)
Zn(1)-N(1)	2.1127(19)	Zn(1)-N(2)	2.095(2)		
O(1)-Zn(1)-O(7)	120.27(8)	O(1)-Zn(1)-O(4)	90.39(7)	O(7)-Zn(1)-O(4)	91.23(7)
O(1)-Zn(1)-N(2)	120.65(7)	O(7)-Zn(1)-N(2)	119.06(9)	N(2)-Zn(1)-O(4)	89.81(7)
N(1)-Zni(1)-O(1)	94.81(6)	O(7)-Zn(1)-N(1)	95.65(7)	N(1)-Zn(1)-O(4)	167.79(8)
N(1)-Zn(1)-N(2)	78.04(7)				
**4**					
Cd(1)-O(1)	2.3964(19)	Cd(1)-O(2)	2.457(3)	Cd(1)-O(4)i	2.474(2)
Cd(1)-O(5)i	2.3476(19)	Cd(1)-O(8)	2.273(2)	Cd(1)-N(1)	2.357(2)
Cd(1)-N(2)	2.363(2)				
O(8)-Cd(1)-O(5)i	100.37(8)	N(1)-Cd(1)-O(8)	91.28(9)	O(5)i-Cd(1)-N(1)	150.14(7)
O(8)-Cd(1)-N(2)	155.26(9)	O(5)i-Cd(1)-N(2)	88.97(7)	N(1)-Cd(1)-N(2)	70.56(8)
O(1)-Cd(1)-O(8)	98.05(8)	O(5)i-Cd(1)-O(1)	78.98(7)	O(1)-Cd(1)-N(1)	126.78(7)
O(1)-Cd(1)-N(2)	106.24(8)	O(8)-Cd(1)-O(2)	89.29(8)	O(2)-Cd(1)-O(5)i	131.90(7)
O(2)-Cd(1)-N(1)	75.07(8)	O(2)-Cd(1)-N(2)	101.51(8)	O(1)-Cd(1)-O(2)	52.96(7)
O(4)i-Cd(1)-O(8)	83.56(8)	O(5)i-Cd(1)-O(4)i	53.88(7)	O(4)i-Cd(1)-N(1)	101.03(8)
O(4)i-Cd(1)-N(2)	83.64(8)	O(4)i-Cd(1)-O(1)	131.99(7)	O(2)-Cd(1)-O(4)i	171.81(10)
**5**					
Mn(1)-O(1)	2.351(4)	Mn(1)-O(2)	2.222(3)	Mn(1)-O(5)	2.212(3)
Mn(1)-O(5)i	2.504(3)	Mn(1)-O(6)i	2.256(3)	Mn(1)-N(1)	2.259(4)
Mn(1)-N(2)ii	2.256(4)				
O(1)-Mn(1)-O(5)i	136.79(12)	O(1)-Mn(1)-O(2)	56.65(13)	O(5)i-Mn(1)-O(2)	166.54(13)
O(6)i-Mn(1)-O(2)	139.39(13)	N(1)-Mn(1)-O(2)	87.70(14)	N(2)-Mn(1)-O(2)	89.50(15)

O(5)-Mn(1)-O(1)	148.80(12)	O(5)-Mn(1)-O(2)	92.16(13)	O(5)-Mn(1)-O(5)i	74.38(12)
O(5)-Mn(1)-O(6)i	128.44(13)	O(5)-Mn(1)-N(1)	89.04(14)	N(2)ii-Mn(1)-O(5)	89.73(15)
O(1)-Mn(1)-O(6)i	82.75(12)	O(5)i-Mn(1)-O(6)i	54.06(11)	N(1)-Mn(1)-O(6)i	92.23(13)
N(1)-Mn(1)-O(1)	90.22(15)	N(1)-Mn(1)-O(5)i	92.48(12)	O(1)-Mn(1)-N(2)ii	89.35(16)
N(2)ii-Mn(1)-O(5)i	89.94(13)	N(2)-Mn(1)-O(6)i	90.77(13)	N(1)-Mn(1)-N(2)ii	176.89(15)
**6**					
Zn(1)-O(1)	1.964(4)	Zn(1)-O(4)	1.922(4)	Zn(1)-N(1)	2.055(4)
Zn(1)-N(2)	2.059(5)				
O(1)-Zn(1)-N(1)	131.3(2)	O(1)-Zn(1)-N(2)	101.6(2)	O(1)-Zn(1)-O(4)	102.8(2)
O(4)-Zn(1)-N(1)	99.62(18)	O(4)-Zn(1)-N(2)	121.1(2)	N(2)-Zn(1)-N(1)	102.71(18)
**7**					
Zni(1)-O(1)	2.038(3)	Zn(1)-O(2)	2.329(4)	Zn(1)-O(5)i	1.939(3)
Zn(1)-N(1)	2.078(5)	Zn(1)-N(2)	2.072(5)		
N(1)-Zn(1)-O(2)	91.35(15)	N(1)-Zn(1)-N(2)	80.23(16)	N(2)-Zn(1)-O(2)	134.90(15)
O(1)-Zn(1)-N(1)	137.38(16)	O(1)-Zn(1)-N(2)	98.07(15)	O(1)-Zn(1)-O(2)	59.76(13)
O(5)i-Zn(1)-N(1)	103.93(15)	O(5)i-Zn(1)-N(2)	129.54(15)	O(5)i-Zn(1)-O(1)	109.11(15)
O(5)i-Zn(1)-O(2)	95.54(14)				
**8**					
Cd(1)-O(1)	2.343(6)	Cd(1)-O(2)	2.348(6)	Cd(1)-O(4)i	2.346(6)
Cd(1)-O(5)ii	2.220(6)	Cd(1)-N(1)	2.300(7)	Cd(1)-N(2)	2.368(7)
N(1)-Cd(1)-N(2)	71.7(2)	N(1)-Cd(1)-O(1)	91.2(2)	N(1)-Cd(1)-O(2)	102.6(3)
N(1)-Cd(1)-O(4)i	91.9(2)	O(1)-Cd(1)-N(2)	156.7(2)	O(1)-Cd(1)-O(2)	55.4(2)
O(4)i-Cd(1)-O(1)	83.4(2)	O(2)-Cd(2)-N(2)	112.0(2)	N(2)-Cd(2)-O(4)i	112.0(2)
O(4)i-Cd(2)-O(2)	136.0(2)	O(5)ii-Cd(2)-N(1)	155.0(2)	O(5)ii-Cd(2)-N(2)	89.7(3)
O(5)ii-Cd(2)-O(1)	110.8(2)	O(5)ii-Cd(2)-O(2)	99.8(2)	O(5)ii-Cd(2)-O(4)i	79.4(2)
**9**					
Cu(1)-O(1)	1.939(2)	Cu(1)-O(4)i	2.184(3)	Cu(1)-O(7)	1.953(2)
Cu(1)-N(1)	2.000(3)	Cu(1)-N(2)	2.019(3)		
O(1)-Cu(1)-O(7)	91.66(10)	O(1)-Cu(1)-N(1)	165.24(12)	N(1)-Cu(1)-O(7)	90.91(11)
N(2)-Cu(1)-O(1)	86.18(10)	N(2)-Cu(1)-O(7)	168.14(12)	N(1)-Cu(1)-N(2)	88.32(11)
O(1)-Cu(1)-O(4)i	99.64(10)	O(7)-Cu(1)-O(4)i	97.70(11)	N(1)-Cu(1)-O(4)i	94.41(11)
N(2)-Cu(1)-O(4)i	94.16(11)				

[a]Symmetry transformations used to generateequivalentatoms:i –*x*+2, –*y*+1, –*z*+1 for **1**; i –*x*+1, –*y*+1, –*z*+1; ii *x*, *y*+1, *z*–1; iii –*x*, –*y*+1, –*z*+1; iv *x*, *y*–1, *z*+2; v *x*, *y*–1, *z*+1for **2**; i –*x*+3/2, *y*, *z*+1/2 for **4**; i –*x*+1, *y*, –*z*+1/2; ii *x*, *y*–1, *z* for **5**; i –*x*+1/2, *y*–1/2, *z*+1/2 for **7**; i –*x*+1/2, *y*–1/2, *z*+1/2; ii –*x*, –*y*+2, *z*+1/2 for **8**; i –*x*+1, –*y*+1, –*z*+1 for **9**.

Table S2. Hydrogen bonds in crystal packing [Å, °] of **1–9**.

Compound	D-H…A	*d*(D-H)	*d*(H…A)	*d*(D…A)	∠DHA	Symmetry code
**1**	O(3)-H(1)···O(2)	0.820	1.879	2.599	145.93	
	O(7)-H(1W)···O(4)	0.880	1.797	2.624	155.58	-*x*+2, -*y*+1, -*z*+1
	O(7)-H(2W)···O(2)	0.796	2.125	2.860	153.63	-*x*+2, -*y*+1, -*z*+2
	O(8)-H(3W)···O(2)	0.842	2.092	2.884	156.62	
	O(8)-H(4W)···O(4)	0.806	1.869	2.674	177.04	-*x*+1, -*y*+1, -*z*+1
**2**	O(3)-H(1)···O(2)	0.820	1.831	2.551	145.64	
	O(6)-H(2)···O(4)	0.820	1.856	2.574	145.49	
	O(9)-H(5)···O(8)	0.820	1.829	2.545	145.08	
	O(12)-H(8)···O(10)	0.820	1.858	2.569	144.38	
**3**	O(3)-H(1)···O(2)	0.820	1.817	2.537	145.72	
	O(6)-H(2)···O(5)	0.820	1.808	2.537	147.25	
	O(7)-H(1W)···O(2)	0.932	1.766	2.681	166.11	-*x*+1, *y*+1/2, -*z*+3/2
	O(7)-H(2W)···O(5)	0.887	1.807	2.640	155.51	
**4**	O(3)-H(1)···O(2)	0.820	1.833	2.555	146.05	
	O(6)-H(2)···O(5)	0.820	1.756	2.505	150.94	
	O(8)-H(1AA)···O(5)	0.871	2.112	2.848	141.78	-*x*+2, -*y*+3/2, *z*+1/2
	O(8)-H(1AB)···O(1)	0.870	1.999	2.752	144.19	*x*+1/2, -*y*+3/2, *z*
**5**	O(3a)-H(3a)···O(1)	0.820	1.906	2.595	141.05	
	O(3b)-H(3b)···O(1)	0.820	1.903	2.619	145.17	
	O(4a)-H(4a)···O(2)	0.820	1.906	2.528	131.83	
	O(4b)-H(4b)···O(2)	0.820	1.907	2.621	144.90	
	O(7a)-H(7a)···O(6)	0.820	1.901	2.607	143.77	
	O(7b)-H(7b)···O(6)	0.820	1.922	2.610	140.85	
**6**	O(3)-H(3)···O(2)	0.820	1.793	2.522	147.28	
	O(6)-H(6)···O(5)	0.820	2.030	2.567	122.60	
**7**	O(3)-H(3)···O(2)	0.820	1.874	2.593	145.61	
	O(6)-H(6)···O(4)	0.820	1.843	2.566	146.35	
**8**	O(3)-H(3)···O(1)	0.820	1.910	2.584	138.89	
	O(6)-H(6)···O(5)	0.820	1.814	2.535	145.86	
**9**	O(3)-H(3)···O(2)	0.820	1.869	2.590	146.02	
	O(6)-H(6)···O(5)	0.820	1.856	2.566	144.26	
	O(7)-H(1W)···O(2)	0.850	1.869	2.719	179.94	
	O(7)-H(2W)···O(5)	0.850	1.735	2.585	179.92	*x*, *y*+1, *z*+1

Table S3. Comparison of related catalytic systems for the Henry reaction between 4-nitrobenzaldehyde and nitroethane.[a]

Entry	Catalyst	Catalyst (mol%)	Solvent	Time (h)	Temp. (˚C)	Product yield (%)	Ref.
1	$[Zn(\mu_2\text{-}H_2L_1)(2,2'\text{-bipy})(H_2O)]_n$ (**3**)	4	CH_3OH	12	70	89	This work
2	$\{[Cu_2(L_a)(H_2O)_2](DMF)_3(H_2O)_4\}_n$ (activated)	5	–	48	50	81	82
3	$\{[Cu(L_b)(DMF)]\cdot DMF\cdot H_2O\}_n$	2	H_2O	40	75	98	83
4	$[Zn(L_c)(H_2O)_2]_n$	3	CH_3OH	48	70	97	85
5	$[Zn(L_d)(H_2O)_2]_n$	3	H_2O	48	70	93	86

[a]Linkers in coordination polymer catalysts: H_4L_a: 5,5′-(piperazine-1,4-diyl)diisophthalic acid; H_2L_b: 5-{(pyridin-4-ylmethyl)-amino} isophthalic acid; H_2L_c: 5-benzamidoisophthalic acid; H_2L_d: 3,3’-{(pyridine-2,6-dicarbonyl)-bis(azanediyl)} dibenzoic acid.

Supporting References

S1. Gu, J. Z.; Cui, Y.; Liang, X. X.; Wu, J.; Lv, D.; Kirillov, A. M. Structurally Distinct Metal-Organic and H-Bonded Networks Derived from 5-(6-Carboxypyridin-3-yl)isophthalic Acid: Coordination and Template Effect of 4,4 '-Bipyridine. *Cryst. Growth Des.* **2016**, *16*, 4658−4670.

S2. Gu, J.-Z.; Wan, S.-M.; Dou, W.; Kirillova, M. V.; Kirillov, A. M. Coordination polymers from an unexplored biphenyl-tricarboxylate linker: hydrothermal synthesis, structural traits and catalytic cyanosilylation. *Inorg. Chem. Front.* **2021**, *8*, 1229−1242.

S3. Gu, J.; Wan, S.; Cheng, X.; Kirillova, M. V.; Kirillov, A. M. Coordination Polymers from 2-Chloroterephthalate Linkers: Synthesis, Structural Diversity, and Catalytic CO_2 Fixation. *Cryst. Growth Des.* **2021**,*21*, 2876−2888.

S4. Gu, J.; Gao, Z.; Tang, Y. pH and Auxiliary Ligand Influence on the Structural Variations of 5(2 '-Carboxylphenyl) Nicotate Coordination Polymers. *Cryst. Growth Des.* **2012**, *12*, 3312−3323.

S5. Gu, J.-Z.; Liang, X.-X.; Cai, Y.; Wu, J.; Shi, Z.-F.; Kirillov, A. M. Hydrothermal assembly, structures, topologies,

luminescence, and magnetism of a novel series of coordination polymers driven by a trifunctional nicotinic acid building block. *Dalton Trans.* **2017**, *46*, 10908−10925.

S6. Ay, U.; Sarli, S. E. Investigation by Fluorescence Technique of the Quenching Effect of Co^{2+} and Mn^{2+} Transition Metals, on Naphthalene-Methyl-Beta-Cyclodextrin Host-Guest Inclusion Complex. *J. Fluoresc.* **2018**, *28*, 1371−1378.

S7. Zhao, H. J.; Zhang, C. Syntheses, Structures, Magnetism and Fluorescence Studies of Two 1D Cu(II) Coordination Polymers Based on Bipyridyl Ligands. *J. Inorg. Organomet. Polym.* **2015**, *25*, 912−920.

S8. Qiao, Y.-F.; Du, L.; Zhou, J.; Hu, Y.; Li, L.; Li, B.; Zhao,Q.-H. Synthesis, structures, and fluorescent properties of azo anthranilic acid and its Cu(II), Co(II), and Ni(II) complexes. *J. Coord. Chem.* **2014**, *67*, 2615−2629.

S9. Zhao, F. F; Dong, H.; Liu, B. B.; Zhang, G. J.; Huang, H.; Hu, H. L.; Liu, Y.; Kang, Z. H. Tuning luminescence *via* transition metal-directed strategy in coordination polymers. *CrystEngComm* **2014**, *16*, 4422−4430.
